# Global Fecal and Plasma Metabolic Dynamics Related to *Helicobacter pylori* Eradication

**DOI:** 10.3389/fmicb.2017.00536

**Published:** 2017-03-30

**Authors:** Theresa Wan-Chen Yap, Alex Hwong-Ruey Leow, Ahmad Najib Azmi, Damien L. Callahan, Guillermo I. Perez-Perez, Mun-Fai Loke, Khean-Lee Goh, Jamuna Vadivelu

**Affiliations:** ^1^Department of Medical Microbiology, Faculty of Medicine, University of MalayaKuala Lumpur, Malaysia; ^2^Department of Medicine, Faculty of Medicine, University of MalayaKuala Lumpur, Malaysia; ^3^Faculty of Medicine and Health Sciences, Universiti Sains Islam MalaysiaKuala Lumpur, Malaysia; ^4^Centre for Chemistry and Biotechnology, School of Life and Environmental Sciences, Deakin UniversityGeelong, VIC, Australia; ^5^Department of Medicine, New York University School of MedicineNew York, NY, USA; ^6^Department of Microbiology, New York University School of MedicineNew York, NY, USA; ^7^Department of Microbiology and Immunology, Yong Loo Lin School of Medicine, National University of SingaporeSingapore, Singapore

**Keywords:** fecal, plasma, *Helicobacter pylori*, lipidomics, metabolomics

## Abstract

**Background:** *Helicobacter pylori* colonizes the gastric mucosa of more than half of the world's population. There is increasing evidence *H. pylori* protects against the development of obesity and childhood asthma/allergies in which the development of these diseases coincide with transient dysbiosis. However, the mechanism underlying the association of *H. pylori* eradication with human metabolic and immunological disorders is not well-established. In this study, we aimed to investigate the local and systemic effects of *H. pylori* eradication through untargeted fecal lipidomics and plasma metabolomics approaches by liquid chromatography mass spectrometry (LC-MS).

**Results:** Our study revealed that eradication of *H. pylori* eradication (i.e., loss of *H. pylori* and/or *H. pylori* eradication therapy) changed many global metabolite/lipid features, with the majority being down-regulated. Our findings primarily show that *H. pylori* eradication affects the host energy and lipid metabolism which may eventually lead to the development of metabolic disorders.

**Conclusion:** These predictive metabolic signatures of metabolic and immunological disorders following *H. pylori* eradication can provide insights into dynamic local and systemic metabolism related to *H. pylori* eradication in modulating human health.

## Introduction

*Helicobacter pylori* is a spiral-shaped, microaerophilic, Gram-negative bacterium that colonizes the gastric mucosa of more than half of the world's population in which its prevalence is as high as 80% in some developing countries (Clyne et al., 2007). Most *H. pylori* carriers remain asymptomatic in their lifetimes, however, a minority group develops peptic ulcers (10–20%), gastric cancer (1–2%), and rarely mucosa-associated lymphoid tissue (MALT) lymphoma (Kusters et al., 2006) later in life. *H. pylori* is believed to have colonized the human stomach even prior to the initial migration of our ancestors out of East Africa at least 100,000 years ago (Moodley et al., 2012). It has been postulated that *H. pylori* may be part of the human indigenous microbiota. However, *H. pylori* is gradually disappearing from the human indigenous microbiota due to development of socioeconomic, modern hygienic practices and advent of antibiotics. There is an growing epidemiological and experimental evidences on the protective effects of *H. pylori* against the development of obesity (Nwokolo et al., 2003; Osawa, 2008; Francois et al., 2011; Yap et al., 2015, 2016), childhood asthma (Chen and Blaser, 2008), allergies (Amberbir et al., 2011), inflammatory bowel diseases (Amnon and Robert, 2012), in which the development of these diseases coincide with transient dysbiosis. However, the underlying protective mechanism of *H. pylori* against the development of these metabolic and immunological disorders is still not well-established.

Over the last two decades, systems biology has emerged as an important tool to provide insights into the role of mammalian gut microbial metabolic interactions in influencing an individual's susceptibility to health and disease outcomes (Martin et al., 2012). The emergence of systems biology coincides with the completion of the Human Genome Project (HGP) (Lander et al., 2001; Venter et al., 2001) and the concomitant emergence of “omics technologies,” namely transcriptomics (Schena et al., 1995; Lashkari et al., 1997), proteomics (Patterson and Aebersold, 2003), metabolomics (Oliver et al., 1998; Fiehn, 2001) and most recently, lipidomics (Han and Gross, 2003; Wenk, 2005). Lipidomics is the global characterization of the structure and function of lipids within a living system (Harkewicz and Dennis, 2011). Omics technologies could be utilized to decipher the vastly complex metabolic exchange between various biological compartments of the *H. pylori* infected human host (including tissues, organs and systemic biofluids) and his gut microbiota and ultimately to help further understand the profound influence exerted by *H. pylori* and gut bacterial microbiota on the metabolic equilibrium of the host and, as a consequence, on his health status.

The present study was carried out in the Malaysian population to determine the implications of *H. pylori* eradication, particularly in the Asian community. We hypothesized that eradication of *H. pylori* (loss of *H. pylori* and/or *H. pylori* eradication therapy) may have undesirable implications on metabolic and immunological disorders. Our earlier studies indicate that *H. pylori* eradication could result in perturbation of gut microbiome (Yap et al., 2016) and homeostasis of human metabolic hormones involved in appetite-control and energy metabolism (Yap et al., 2015). This may eventually lead to the development of metabolic disorders. In this study, we aimed to investigate the association of *H. pylori* eradication with human metabolic and immunological disorders through untargeted fecal lipidomics and plasma metabolomics approaches by liquid chromatography mass spectrometry (LC-MS). Fecal lipidomics enabled us to evaluate the local effects following *H. pylori* eradication on the gut microbiota, whereas plasma metabolomics reflected the systemic effects of *H. pylori* eradication.

## Materials and methods

### Study population

This study is part of the presently on-going ESSAY (Eradication Study in Stable Adults/Youths) study in New York, the European Center, and Malaysia. The recruitment of volunteers in Malaysia was conducted at the University of Malaya Medical Centre (UMMC) between June 2012 and May 2014 as described in details in our early study (Yap et al., 2015). Firstly, healthy young adults between the ages of 18 and 30 years old were assessed for eligibility. The exclusion criteria for the study were diabetes, hyper or hypothyroidism, prior gastric or bariatric surgery, prior documented treatment of *H. pylori*, antibiotic use within 4 weeks of enrollment, steroid or other immunomodulating drugs use within 4 weeks of enrolment, recent vaccination and Charlson weighed comorbidity index <2. The study protocol was reviewed and approved by Medical Ethics Committee at UMMC (Ref No. 877.1). Prior to study participation, written informed consent was obtained from qualified volunteers. *H. pylori*'s status of the qualified candidates was determined as described earlier (Yap et al., 2015).

### Sample collection

**Figure 1:**
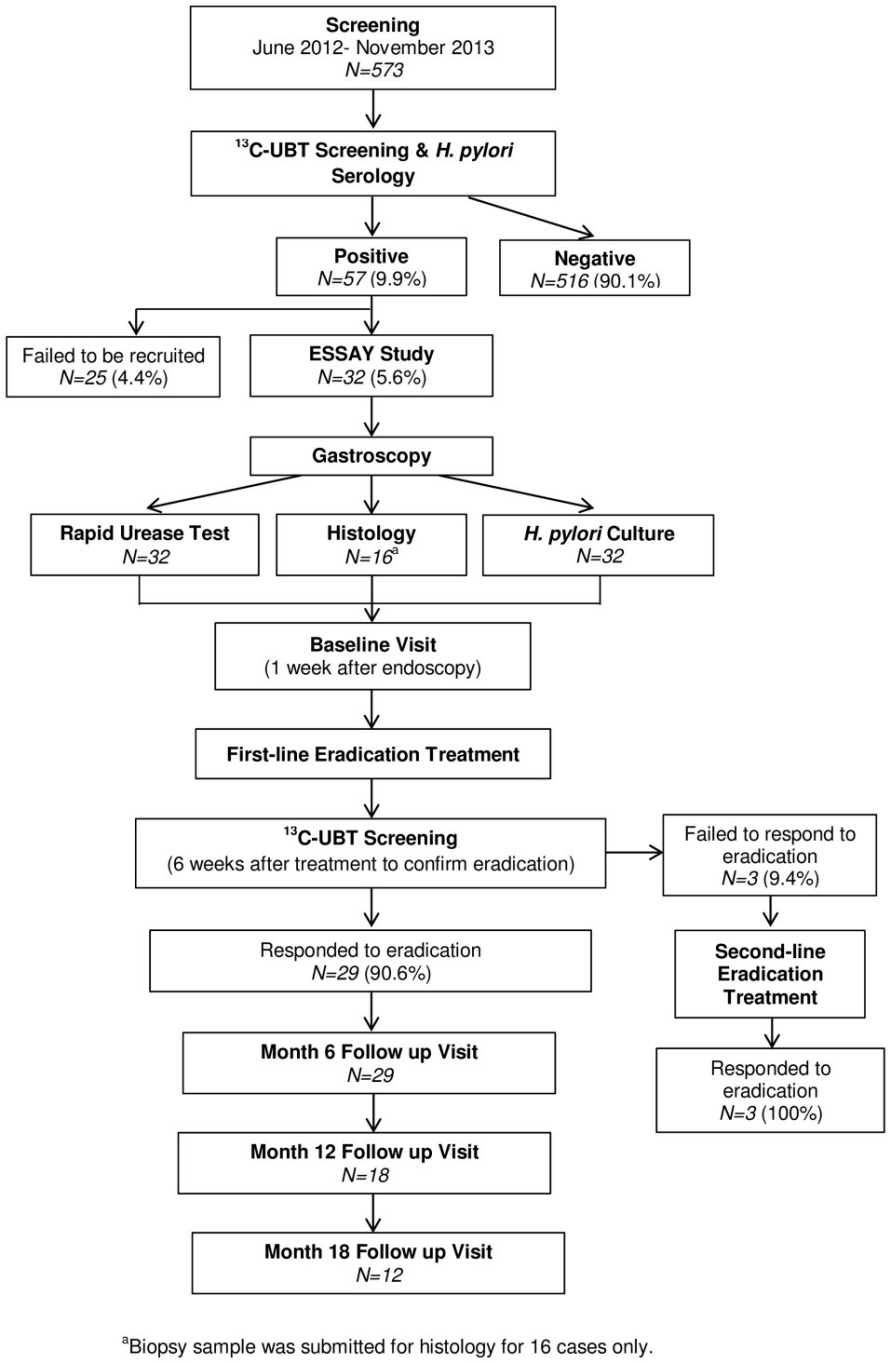
**Summary of ESSAY Study (Yap et al., 2015)**.

Sample collection was performed as reported in a previous investigation using the same study population (Yap et al., 2015). Figure 1 illustrates the workflow for enrolment of *H. pylori*-positive volunteers into the ESSAY Study. As reported previously, 57 (9.9%) of the 573 volunteers screened in the ESSAY study were tested positive for *H. pylori* using both non-radioactive ^13^C Urea Breath Test (UBT) and detection of anti-*H. pylori* antibodies and were considered as *H. pylori*-positive. However, only 32 agreed and consented to participate in the study (Yap et al., 2015).

### Baseline visit

*H. pylori*-positive volunteers were instructed to arrive at the Endoscopic Unit of UMMC at 8 a.m. after 12 h of fasting. 5 ml of fasting blood was collected in EDTA-coated tube. The protease inhibitor, 4-(2-Aminoethyl) benzenesulfonyl fluoride hydrochloride (AEBSF) (Sigma-Aldrich, St. Louis MO), at a concentration of 1 mg/ml, was added to the blood collection tubes. Tubes were kept on ice until centrifuged, and plasma was kept at −80°C for metabolomics analysis. Fecal samples were also collected and frozen immediately at −80°C for lipidomics analysis.

### *H. pylori* eradication therapy

Volunteers who tested positive for *H. pylori* were prescribed with a 7-day twice daily regimen and a proton pump inhibitor as per current standard of care (amoxicillin 1,000 mg, clarithromycin 500 mg, and pantoprazole 40 mg). ≥6 weeks after completion of the treatment protocol, *H. pylori* eradication was ascertained using the non-radioactive ^13^C Urea Breath Test. Volunteers who failed the first-line eradication regimen were offered second-line eradication therapy with a 2-week twice daily regime (amoxicillin 1,000 mg, levofloxacin 500 mg and rabeprazole 20 mg) (Yap et al., 2015).

### Follow-up assessment

Eventually, 29 volunteers whom *H. pylori* infection were successfully eradicated were recruited to the ESSAY Study. These volunteers returned for follow-up assessment at 6, 12, and 18 months, paralleling that described for the baseline visit. The number of volunteers returned for follow-up assessment was 29, 18, and 12 respectively (Figure 1).

### Fecal sample preparation and lipids extraction

All the organic solvents used for fecal lipids and plasma metabolites extraction were of HPLC-grade. Methanol, acetonitrile and isopropanol were purchased from Friendemann Schmidt (Australia), methyl *tert*-butyl ether/hexafluoroisopropanol (MTBE) was purchased from Sigma-Aldrich (St. Louis, MO), LCMS-grade water from a Milli-Q water purification system (EMD Millipore, Billerica, MA) and formic acid from Sigma-Aldrich (St. Louis, MO).

Firstly, fecal slurry for each fecal sample was prepared by combining 150 mg solid fecal material in three ml of water. The fecal slurry was then diluted with water in 1:3 ratio. The diluted fecal slurries were then pooled into different groups according to gender, body mass index (BMI), and race (Table S1) to ascertain whether these variables are the confounding factors of this study. 50 μl of each diluted fecal slurry samples from different group was placed into 2 ml centrifuge tube. Five hundred microliters of methanol was added to each tube and vortexed for 30 s to ensure all components were thoroughly mixed. The mixed samples were then vortexed intermittently for 15 min and centrifuged at 12,000 × g for 10 min to pellet insoluble fecal material. The supernatant was then transferred to 15 ml polypropylene centrifuge tubes. MTBE, 2 ml per sample, was added to each tube, vortexed to mix, and incubated at room temperature for 10 min to precipitate proteins. The lipid-containing supernatants were transferred to new 15 ml tubes, and 1.5 ml of water was added to induce phase separation. Samples were vortexed 30 s to mix, then centrifuged at 5,000 × g for 5 min. After phase separation, the lipid-containing MTBE phase forms the top layer, whilst the methanol and water forms the bottom layer. The top layer lipid extracts were aspirated and transferred to new tubes (Gregory et al., 2013). The lipid extracts were dried in a CentriVap Concentrator Systems (LABCONCO, Kansas City, MO) at 4°C and then resuspended to a 500 μl of acetonitrile:isopropanol:water (65:30:5), vortexed for 30 s and then centrifuged again at 12,000 × g for 5 min before injecting into liquid chromatography system.

### Plasma sample preparation and metabolites extraction

Prior to the experimental sample extraction, the ratio of methanol to sample (v/v) for plasma metabolite extraction as well as the resuspension volume of 95:5 water:acetonitrile were optimized. Similar to fecal lipids extraction, the experimental plasma samples were pooled into different groups according to gender, body mass index (BMI), and race (Table S1) to ascertain whether these variables are the confounding factors of this study. Protein precipitation was conducted by adding 500 μl aliquots of ice cold methanol to 100 μl aliquots of plasma samples. The samples were instantaneously vortexed for 30 s and incubated on ice for 20 min. After centrifugation at 12,000 × g for 10 min, the metabolite containing supernatant was removed from the precipitated protein pellet and transferred to fresh tubes. The supernatant samples were dried in a CentriVap Concentrator Systems (LABCONCO, Kansas City, MO) at 4°C and then resuspended to a 100 μl of water: acetonitrile (95:5), vortexed for 30 s and then centrifuged again at 12,000 × g for 5 min before injecting into liquid chromatography system (Denery et al., 2010).

### Fecal lipidomics and plasma metabolomics analysis

#### High-performance liquid chromatography mass spectrometry (LC-MS)

LC-MS analyses on the fecal lipids were performed on a 1260 Infinity High Performance Liquid Chromatography system coupled with a 6540 UHD Accurate-Mass Q-TOF mass spectrometer from Agilent Technologies (Santa Clara, CA) with a Dual Agilent Jet Stream Electrospray Ionization (Dual AJS ESI) source.

Analysis was performed utilizing both positive ionization and negative ionization mode of the Dual AJS ESI source and using All Ions MS/MS technique. All Ions MS/MS alternates between high and low energy scans during acquisition: high energy scans create fragment ions, low energy scans preserve the precursor ions. Optimization of several LC parameters such as injection volume, flowrate, and LC gradient were conducted. To ensure the reproducibility and robustness of the data acquired, a pooled biological quality control samples (PBQC) (constituted of pooled aliquots of all experimental fecal samples) and a lipid standards mixture (Avanti Polar Lipids, Alabaster, AL) (Table S2) were periodically injected throughout the duration of analysis. The sample run order was also assigned randomly to avoid any systematic bias.

The aqueous mobile phase (mobile phase A) was 60% acetonitrile and 40% water with 10 mM ammonium acetate (ThermoFisher Scientific, Waltham, MA) and the organic mobile phase (mobile phase B) was 10% acetonitrile and 90% isopropanol with 10 mM ammonium acetate. A 10 μl of extracted fecal lipids was loaded onto Zorbax Eclipse Plus C18, 2.1 × 100 mm, 1.8 μm reverse phase column (Agilent Technologies, Santa Clara, CA) with 70% mobile phase B at 0.20 ml/min. Lipids were eluted from the column with a gradient of 70–100% mobile phase B over 8 min at 0.20 ml/min followed by a 7 min rinse of 100% mobile phase B. The column was immediately re-equilibrated under the initial conditions (isocratic hold at 70% mobile phase B) for 10 min.

Consistent mass accuracy (<2 ppm) was maintained through a constant infusion (2 μl/min) of reference calibrants, methyl stearate and HP-122l (with reference mass of 299.294457 and 1221.990637 m/z, respectively), via a reference nebulizer. Data were collected in both positive and negative ESI mode acquiring in centroid mode from 100 to 1,700 m/z with an acquisition rate of 1 spectrum per second in 2 GHz extended dynamic range. The Dual AJS ESI capillary voltage was set at 3.5 kV and nozzle voltage was set at 1 kV; The gas temperature, drying gas flow, nebulizer pressure, sheath gas temperature and sheath gas flow were set at 300°C, 8 l/min, 35 psig, 350°C and 11 l/min, respectively. The fragmentor and skimmer voltage were set at 175 and 65 V, correspondingly. For All Ions MS/MS, collision energy was ramped from 0 to 40 V during each 4 s data collection cycle. All the acquired mass spectral data was collected in a .*d* format.

The platform and methodology for LC-MS analyses on the plasma metabolomics were the same as fecal lipids mentioned above except that instead of using All Ions MS/MS technique, only MS was used. A PBQC (constituted of pooled of all experimental plasma samples) and an external commercial Waters MetID Small Molecule Standard Mix (constituted of 5-hydroxy omeprazole and omeprazole sulfone) (Waters Corporation, Milford, MA) were periodically injected throughout the duration of analysis.

For positive ionization, the aqueous mobile phase (mobile phase A) was water with 0.1% formic acid and the organic mobile phase (mobile phase B) was acetonitrile with 0.1% formic acid. For negative ionization, the aqueous mobile phase (mobile phase A) was water with 1 mM ammonium fluoride (Sigma-Aldrich, St. Louis, MO) and the organic mobile phase (mobile phase B) was acetonitrile only. The instrument control and data acquisition were conducted using the MassHunter Workstation Data Acquisition software, version B.05.01 (Agilent Technologies, Santa Clara, CA). A five μl of extracted plasma metabolites was loaded onto Zorbax Eclipse Plus C18, 2.1 × 100 mm, 1.8 μm reverse phase column (Agilent Technologies, Santa Clara, CA) with 5% mobile phase B at 0.45 ml/min. Metabolites were eluted from the column with a gradient of 5–100% mobile phase B over 22 min at 0.45 ml/min followed by a 5 min rinse of 100% mobile phase B. The column was immediately re-equilibrated under the initial conditions (isocratic hold at 5% mobile phase B) for 5 min.

Data were collected in both positive and negative ESI mode acquiring in profile mode from 70 to 1,700 m/z with an acquisition rate of 2 spectra per second in 2 GHz extended dynamic range. The Dual AJS ESI capillary voltage was set at 3 kV; The gas temperature, drying gas flow, nebulizer pressure, sheath gas temperature and sheath gas flow were set at 300°C, 10 l/min, 45 psig, 250°C, and 8 l/min, respectively. For the MS Q-TOF, the fragmentor and skimmer voltage were set at 140 and 60 V, correspondingly. All the acquired mass spectral data was collected in a .*d* format.

#### Data processing and statistical analysis

Molecular features were extracted (Find by Molecular Feature) with Agilent MassHunter Workstation Qualitative Analysis software, version B.06.00 (Agilent Technologies, Santa Clara, CA). A feature condenses the abundances from all the specified adducts and isotopes of a compound into a single compound. The extracted data was exported as compound exchange file (.cef files) and imported into Mass Profiler Professional (MPP) software, version 12.1 (Agilent Technologies, Santa Clara, CA) for the first round of data filtering, alignment and normalization. The generated list of compounds was then exported as a recursive list. Subsequently, a targeted feature finding (Find by Formula-Options) was performed with MassHunter Workstation Qualitative Analysis software by using the recursive list as source of formula to confirm the identified compounds; a mass tolerance of 5 ppm was set. The recursed data was then exported as compound exchange file (.cef files). The recursed .cef files were then imported into MPP to perform data processing, compounds identification and annotation, and differential and statistical analysis between baseline and post-*H. pylori* eradication groups. MPP integrated with ID Browser was used for identification and annotation of compounds using LC/MS Personal Compound Databases (METLIN database). A one-way ANOVA and Tukey's honest significant difference (HSD) *post-hoc* analysis were performed to compare the lipids/metabolites between Baseline vs. 6 months, Baseline vs. 12 months post-*H. pylori* eradication and Baseline vs. 18 months post-*H. pylori* eradication; a two-tailed *p* < 0.01 and false discovery rate (FDR) of <1% were considered significant. A multivariate statistical analysis, i.e., Principle Component Analysis (PCA) and Hierarchical Cluster Analysis (HCA), were performed to observe clustering, trends and outliers in our data sets and to examine whether the Baseline and post-*H. pylori* eradication groups could be differentiated using lipid/metabolite profiles. For HCA analysis, clustering algorithm base on Euclidean distance was used. Identifying small molecules/lipids that showed significant changes between Baseline and post-eradication groups is the most laborious and time-consuming aspect of non-targeted metabolomics/lipidomics. Each identified entities were manually checked through to filter out wrongly annotated or ambiguous lipids as well as non-annotated entities and sorted them into different categories. The significant differentially expressed lipids/metabolites were then mapped to pathways using Pathway Analysis integrated in MPP.

## Results

### Fecal lipidomics profiling

The LC-MS approach was used to investigate the dynamics of fecal lipidomics profiles pre- and post-*H. pylori* eradication on samples collected at Baseline prior to *H. pylori* eradication and 6, 12, and 18 months post-eradication. The numbers of fecal samples that were collected in the study were 29, 23, 14, and 11 samples respectively. In order to minimize bias as well as to identify confounding factors that may affect our findings, the collected fecal samples were pooled into different groups according to gender, body mass index (BMI), and race for LC-MS analysis (Table S1).

The LC-ESI-TOF total ion chromatograms (TIC) of fecal lipidomics for Baseline and post-eradication groups, acquired under ESI positive and negative ionization, correspondingly, demonstrated several peaks with different intensities, marked by asterisks in the TIC, that were readily observed between the Baseline and post-*H. pylori* eradication groups (Figures S1, S2). Data analysis using one-way ANOVA and Tukey's honest significant difference (HSD) *post-hoc* analysis of the fecal lipidome revealed a large number of significantly changed molecular features between Baseline and post-*H. pylori* eradication groups and a total of approximately 1,300–1,600 molecular features were extracted from a typical positive and negative LC-ESI-TOF chromatogram between Baseline and post-*H. pylori* eradication groups (Table S3). Of the total molecular features, 513 and 434 of these features were significantly changed between Baseline and post-eradication groups, acquired under positive and negative ionization respectively.

**Figure 2:**
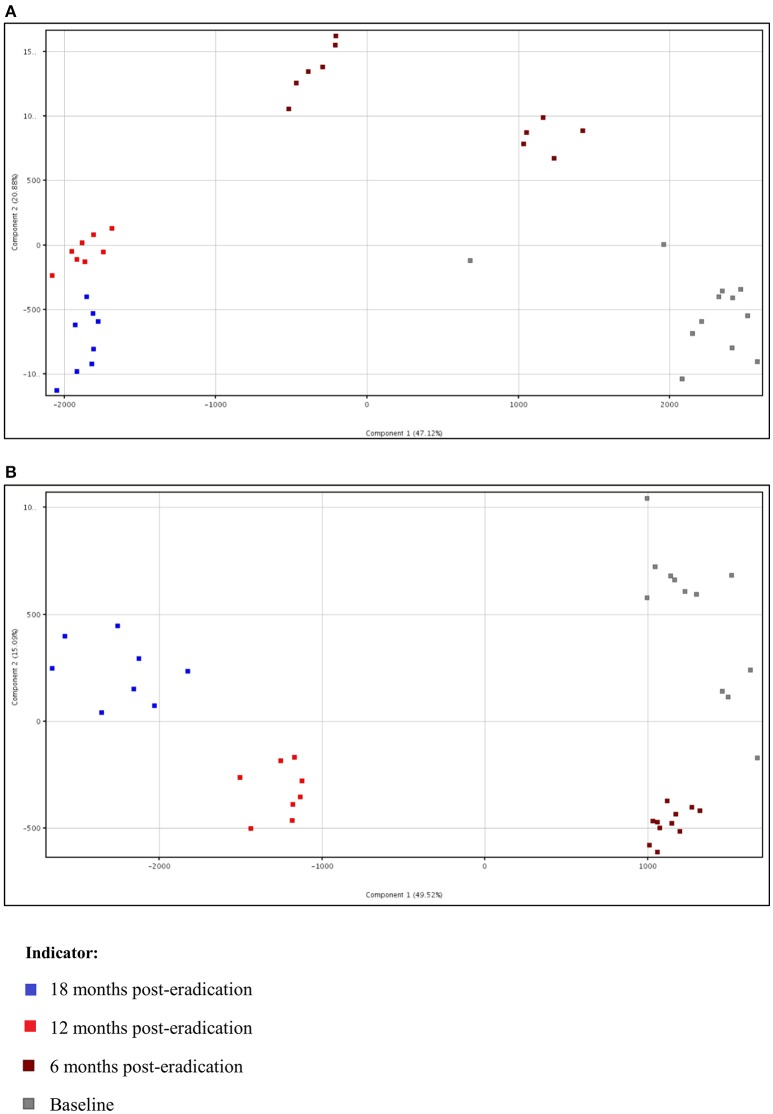
**Two-dimensional PCA scores plot of fecal lipidomics for Baseline, 6, 12, and 18 months post-***H. pylori*** eradication, acquired under both (A)** positive and **(B)** negative ionization modes.

**Figure 3:**
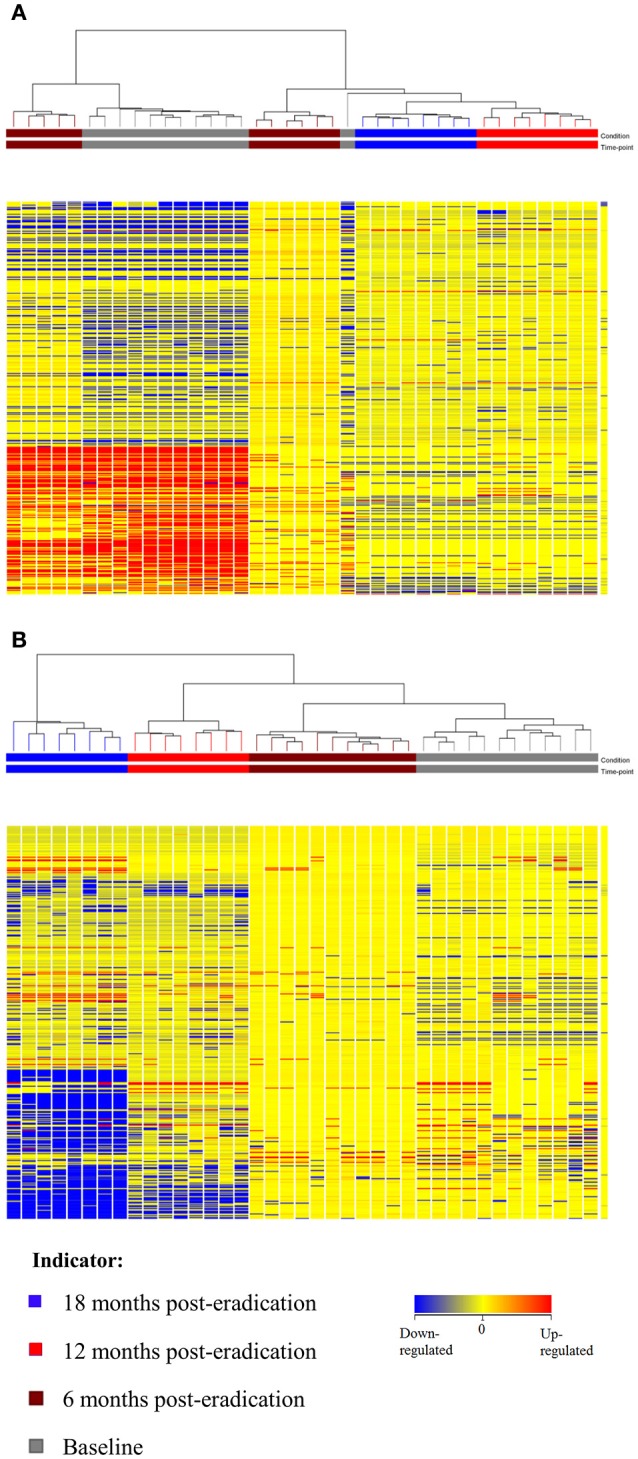
**HCA dendrogram of fecal lipidomics for Baseline, 6, 12, and 18 months post-***H. pylori*** eradication, acquired under both (A)** positive and **(B)** negative ionization modes.

Subsequently, a multivariate statistical principal component analysis (PCA) and hierarchical cluster analysis (HCA) were employed to examine whether samples belonging to Baseline and post-eradication groups could be differentiated based on their lipids profiles. Both the two-dimensional PCA scores plots and HCA dendrograms showed clear separation of samples of Baseline and post-*H. pylori* eradication groups, under both positive and negative ionization modes (Figures 2, 3). However, these plots did not show distinct separation between the different pooled fecal groups which indicated that gender, BMI and race were not the confounding factors in this study.

### Identification of potential fecal lipids

In order to identify the potential fecal lipids associated with *H. pylori* eradication, the significantly altered molecular features were identified and annotated as entities using Mass Profiler Professional (MPP) integrated with ID Browser and LC/MS Personal Compound Databases (METLIN database). In brief, 161, 271, and 269 entities were significantly changed when we compared Baseline vs. 6 months, Baseline vs. 12 months, and Baseline vs. 18 months post-*H. pylori* eradication, respectively (File S1). After excluding the redundancies (e.g., lipids detected in both ionization modes), it was found that when Baseline was compared with 6 months-post eradication, 34 and 13 lipids were up-regulated and down-regulated respectively. Conversely, more lipids were down-regulated when Baseline was compared to the 12 and 18 months-post eradication groups. When Baseline was compared with 12 months-post eradication, 29 and 43 lipids were up-regulated and down-regulated respectively. When Baseline was compared with 18 months-post eradication, 25 and 45 lipids were up-regulated and down-regulated correspondingly (Table S4). These lipids could be categorized into key lipid classes such as fatty acyls, glycerolipids, glycerophospholipids, sphingolipids, sterol lipids, prenol lipids and saccharolipids, of which five were mapped to different biochemical pathways which include retrograde endocannabinoid signaling and sphingolipid metabolism (Table 1).

**Table 1:** **Selected significantly expressed fecal lipids (***p*** < 0.001) between Baseline and post-***H. pylori*** eradication groups**.

**KEGG/HMDB/LMID**	**Lipid**	**Class**	**Pathway**	**m/z**	**RT (min)**	**Comparison of expression level at 6 months post-eradication to Baseline^a^**	**Comparison of expression level at 12 months post-eradication to Baseline^a^**	**Comparison of expression level at 18 months post-eradication to Baseline^a^**
C19913/LMFA08020002	Anandamide 0-phosphate	Fatty acyls	Retrograde endocannabinoid signaling	445.2819	14.79	+11.72	+3.40	−3.31
C00836/	Sphinganine	Sphingolipids	Sphingolipid metabolism	302.3567	1.56	+1.77	+1.30	+0.82
HMDB00269/LMSP01020001								
C12144/	Phytosphingosine	Sphingolipids	Sphingolipid metabolism	318.2937	1.45	−19.48	−18.77	−4.25
HMDB04610/LMSP01030001								
HMDB13244/LMSL01040002	Lipid A -disaccharide-1-P	Saccharolipids	−	661.4385	10.03	+0.38	+3.98	+16.15
LMPR04000022	32,35-anhydrobacteriohopaneterol	Prenol lipids	−	527.4471	11.54	+0.25	+0.21	−13.32

a *Expression level indicates log_2_ fold change (FC). + indicates up-regulated in post-eradication group; − indicates down-regulated in post-eradication group*.

### Plasma metabolomics profiling

LC-MS was also used for untargeted plasma metabolomics to investigate the systemic changes in small molecules that may be associated with *H. pylori* eradication on the same study population. Plasma metabolomics profiling was performed on samples collected at Baseline, 6, 12, and 18 months-post *H. pylori* eradication. The number of plasma samples collected at Baseline, 6, 12, and 18 months-post *H. pylori* eradication were 29, 29, 18, and 12 samples respectively. Parallel to fecal lipidomics study, the collected plasma samples were pooled into different groups according to gender, body mass index (BMI), and race for LC-MS analysis (Table S1).

Several dominant differences of peak intensities, marked by asterisks in the TIC, could be noted from the LC-ESI-TOF TIC of plasma metabolites of Baseline and post-eradication groups, acquired under ESI positive and negative ionization (Figures S3, S4). One-way ANOVA and Tukey's HSD *post-hoc* analysis showed that approximately a total of 7,000–20,000 molecular features could be extracted from a typical positive LC-ESI-TOF chromatogram whereas only approximately 2,000–5,000 features could be extracted from a typical negative LC-ESI-TOF chromatogram (Table S5). Of this enormous amount of molecular features detected, only 5,932 and 1,867 features were markedly changed between Baseline and post-eradication groups, acquired under positive and negative ionization respectively.

**Figure 4:**
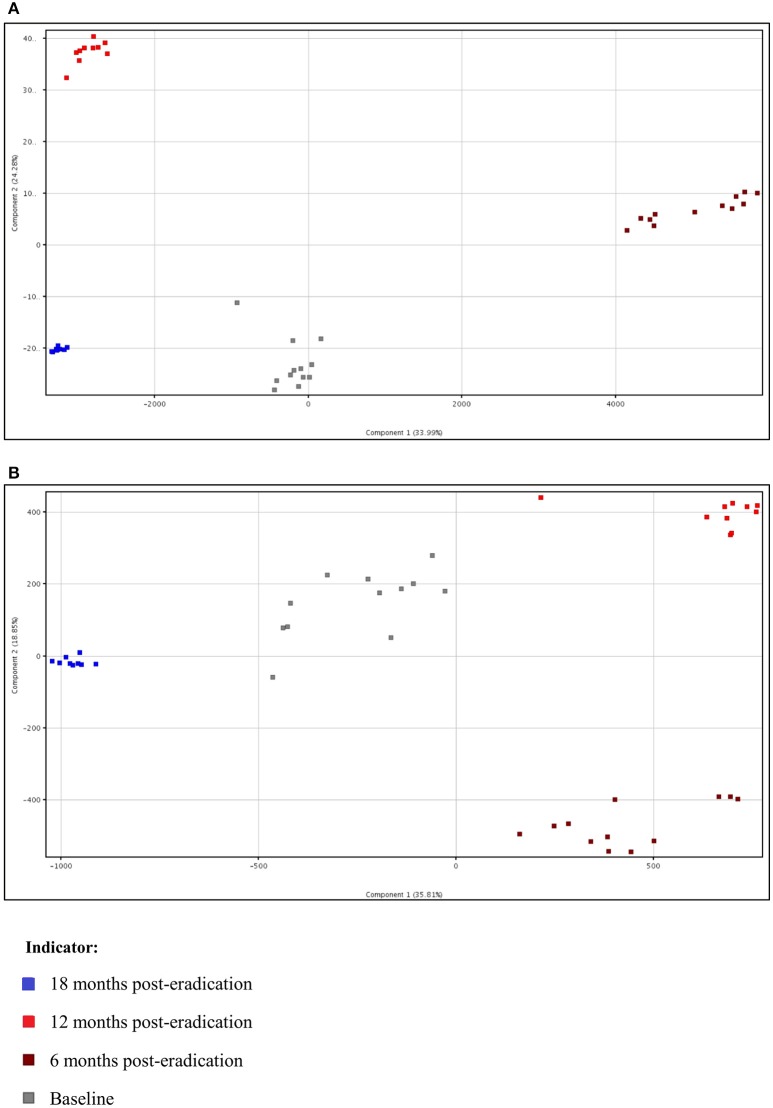
**Two-dimensional PCA of plasma metabolomics for Baseline, 6 months, 12 months, and 18 months post-***H. pylori*** eradication, acquired under both (A)** positive and **(B)** negative ionization modes.

**Figure 5:**
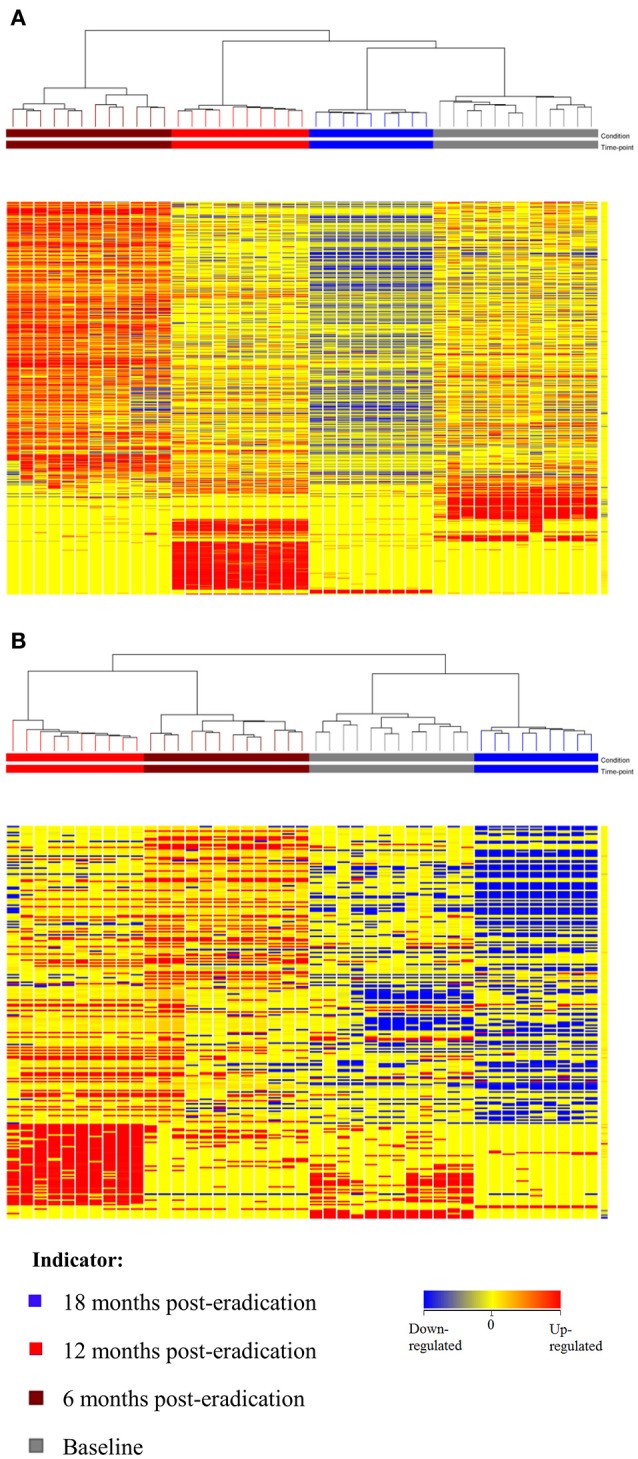
**HCA dendrogram of plasma metabolomics for Baseline, 6, 12, and 18 post-***H. pylori*** eradication, acquired under both (A)** positive and **(B)** negative ionization modes.

Subsequently, a multivariate statistical principal component analysis (PCA) and hierarchical cluster analysis (HCA) were employed to examine whether samples belonging to Baseline and post-eradication groups could be differentiated based on their metabolite profiles. Both of these plots also showed clear separation of samples between the Baseline and post-*H. pylori* eradication groups, under both positive and negative ionization modes, as illustrated in Figures 4, 5. Parallel to fecal lipidomics analysis, both the two-dimensional PCA scores plots and HCA dendrograms did not show distinct separation between the different pooled plasma groups which indicated that gender, BMI and race were not the confounding factors.

### Identification of potential small molecules in plasma

The same identification and annotation approaches were used for both lipid and metabolite datasets. Additionally, for plasma metabolomics, exogenous metabolites and xenobiotics were also filtered out as we were only interested in investigating the effects of *H. pylori* eradication on the human endogenous plasma metabolomes. To sum up, a total of 1,080, 1,125, and 1,262 entities were significantly changed for comparison of Baseline vs. 6 months, Baseline vs. 12 months, and Baseline vs. 18 months post-*H. pylori* eradication, respectively (File S2).

As mentioned above, although the same metabolite could be identified by the ID Browser as several different entities, are actually the components of one compound. After removing the redundant compounds, it was found that when the Baseline samples were compared with 6 months-post eradication, there were 222 and 348 metabolites that were up-regulated and down-regulated respectively. When Baseline was compared with 12 months-post eradication, the up- and down-regulated metabolites were 239 and 359 correspondingly. Conversely, only 45 metabolites were up-regulated but there were 551 metabolites down-regulated when Baseline was compared with 18 months-post eradication group (Table S6). These significantly changed metabolites included amino acids, purines, pyrimidines, cofactors, polar lipids etc. Among these significantly expressed metabolites, 71 of them could be categorized into 22 major classes of metabolites and mapped to different biochemical pathways (Table 2). From the Pathway Analysis, porphyrin and chlorophyll metabolism was found to be the major perturbed pathway followed by tryptophan metabolism and bile acid biosynthesis.

**Table 2:** **Selected significantly expressed plasma metabolites (***p*** < 0.001) between Baseline and post-***H. pylori*** eradication groups**.

**KEGG/ HMDB/ LMID**	**Metabolite**	**Class**	**Pathway**	**m/z**	**RT (min)**	**Comparison of Expression level at 6 months post- eradication to Baseline^a^**	**Comparison of Expression level at 12 months post- eradication to Baseline^a^**	**Comparison of Expression level at 18 months post- eradication to Baseline^a^**
C00345/HMDB01316	6-Phosphogluconic acid	Carbohydrates and carbohydrate conjugates	Pentose Phosphate Pathway	139.0196	3.12	+13.24	−	−
C01598/HMDB01389	Melatonin	Indoles and derivatives	Tryptophan metabolism Melatonin biosynthesis	233.1282	5.00	−10.26	−12.06	−12.06
C01017/HMDB00472	5-Hydroxytryptophan	Indoles and derivatives	Tryptophan metabolism Melatonin biosynthesis	221.0916	1.70	−1.14	−6.60	−9.78
C00463/HMDB00738	Indole	Indoles and derivatives	Tryptophan metabolism	116.0507	2.13	+2.19	+1.07	−7.52
C05635/HMDB00763	5-Hydroxyindoleacetic acid	Indoles and derivatives	Tryptophan metabolism	190.0509	1.21	+1.10	−4.38	−13.99
C00328/HMDB00684	Kynurenine	Benzene and substituted derivatives	Tryptophan metabolism	209.0918	1.43	−5.78	+2.60	−15.71
C05647/HMDB12948	Formyl-5-hydroxykynurenamine	Benzene and substituted derivatives	Tryptophan metabolism	207.0770	1.22	+2.72	+0.912	−15.74
C01829/HMDB00248	Thyroxine	Benzene and substituted derivatives	Tyrosine metabolism Thyroid hormone synthesis	777.6942	12.64	+2.39	−0.43	−9.25
C15522/HMDB02281	4a-Hydroxytetrahydrobiopterin	Pteridines and derivatives	Tryptophan metabolism Phenylalanine and tyrosine metabolism	302.0840	5.93	−14.40	−7.80	−18.57
C09332/HMDB06825	Tetrahydrofolyl-[Glu](2)	Pteridines and derivatives	Folate biosynthesis	597.2033	3.21	+16.98	+11.44	−8.54
C05843/HMDB04194	N-Methyl-4-pyridone-3-carboxamide	Pyridines and derivatives	Nicotinate and nicotinamide metabolism	153.0653	0.96	+2.96	−8.30	−8.30
C00791/HMDB00562	Creatinine	Azolines	Arginine and proline metabolism	112.0517	0.67	+2.26	−0.57	−19.87
C00262/HMDB00157	Hypoxanthine	Imidazopyrimidines	Purine metabolism	137.0452	0.80	−2.69	+10.19	−4.32
C00147/HMDB00034	Adenine	Imidazopyrimidines	Purine metabolism	134.0471	0.93	+3.20	−14.88	−14.88
C04376/HMDB01308	5′-Phosphoribosyl-N-formylglycinamide (FGAR)	Glycinamide Ribonucleotides	Purine metabolism	332.0851	12.29	−13.98	−9.94	−18.75
C01261/HMDB01340	P1,P4-Bis(5′-guanosyl) tetraphosphate (GppppG)	(5′->5′)- dinucleotides	Purine metabolism	869.0477	14.65	−13.65	−6.68	−11.45
C00366/HMDB00289	Uric acid	Alkaloids and derivatives	Purine metabolism	167.0212	0.59	+3.56	+1.92	−17.50
C02067/HMDB00767	Pseudouridine	Nucleoside and nucleotide analogs	Pyrimidine metabolism	243.0624	0.76	+3.00	+1.64	+1.64
C05983/HMDB06806	Propinol adenylate	Purine nucleotides	Propanoate metabolism	426.0801	0.62	−1.85	−12.52	−17.24
C02199/HMDB12305	UDP-L-rhamnose	Pyrimidine nucleotides	Amino sugar and nucleotide sugar metabolism	573.0505	0.69	−14.98	−14.98	−14.98
C19972	2,4-Bis(acetamido)-2,4,6-trideoxy-beta- L-altropyranose	−	Amino sugar and nucleotide sugar metabolism	247.1287	2.33	−6.10	−2.55	−15.88
C00158/HMDB00094	Citric acid	Carboxylic acids and derivatives	Tricarboxylic acid cycle (TCA)	215.0154	0.71	+4.41	−3.21	−8.38
C02237/HMDB00805	R-(+)-Pyrrolidone-5-carboxylic acid	Carboxylic acids and derivatives	D-glutamine and D-glutamate metabolism	130.0496	6.13	+3.74	−14.97	−14.97
C11684	MET-Enkephalin	Carboxylic acids and derivatives	Neuroactive ligand-receptor interaction	591.2605	16.84	−6.63	+4.61	−4.97
C04462/HMDB12266	N-Succinyl-2-amino-6- ketopimelate	Carboxylic acids and derivatives	Lysine biosynthesis	285.1320	7.80	+2.53	−5.42	−12.29
C04148/HMDB06344	Alpha-N-Phenylacetyl-L-glutamine	Carboxylic acids and derivatives	Phenylalanine metabolism	265.1183	6.12	+1.65	+11.41	−12.21
C11588	cis-3-(Carboxy-ethyl)-3,5-cyclo-hexadiene-1,2-diol	Carboxylic acids and derivatives	Phenylalanine metabolism	183.0670	1.11	+22.34	+4.13	−1.87
C00746/HMDB02865	Oxytocin	Carboxylic acids and derivatives	cAMP signaling pathway	521.2544	7.79	+8.94	+16.89	+1.99
C03413/HMDB02172	N1,N12-Diacetylspermine	Carboxylic acids and derivatives	Polyamine metabolism	309.2256	9.80	+4.63	+16.58	−
C00016/HMDB01248	Flavin adenine dinucleotide (FAD)	Flavin nucleotides	−	786.1643	6.36	+2.04	−9.58	−9.58
HMDB11612	6-Hydroxy Flavin adenine dinucleotide	Flavin nucleotides	−	819.1830	9.30	+1.34	−1.75	−7.44
C00194/HMDB02086	Adenosylcobalamin	−	Porphyrin and chlorophyll metabolism Riboflavin metabolism	693.3397	18.96	−18.21	−18.21	−18.21
C03262/HMDB01261	Coproporphyrinogen III	Tetrapyrroles and derivatives	Porphyrin and chlorophyll metabolism Heme biosynthesis	329.1504	1.19	+2.57	+2.23	−11.87
C02191/HMDB00241	Protoporphyrin IX	Tetrapyrroles and derivatives	Porphyrin and chlorophyll metabolism Heme biosynthesis	580.2920	17.64	+1.31	+5.32	−6.3
HMDB00668	Hematoporphyrin	Tetrapyrroles and derivatives	Porphyrin and chlorophyll metabolism Heme biosynthesis	599.2856	12.36	+3.58	−5.65	−9.73
HMDB00683	Harderoporphyrin	Tetrapyrroles and derivatives	Porphyrin and chlorophyll metabolism Heme biosynthesis	626.2958	19.40	−6.89	+5.69	−3.25
C00500/HMDB02309	Biliverdin IX	Tetrapyrroles and derivatives	Porphyrin and chlorophyll metabolism	583.2551	14.19	+1.94	−2.85	−10.57
C05793/HMDB04159	L-Urobilin	Tetrapyrroles and derivatives	Porphyrin and chlorophyll metabolism: Bilirubin degradation	595.3480	14.93	+10.82	−3.16	−4.68
C05794/HMDB04160	I-Urobilin	Tetrapyrroles and derivatives	Porphyrin and chlorophyll metabolism: Bilirubin degradation	591.3163	15.08	−0.85	−12.83	−14.40
C05790/HMDB01898	Mesobilirubinogen	Tetrapyrroles and derivatives	Porphyrin and chlorophyll metabolism: Bilirubin degradation	615.3149	15.05	+3.64	−9.60	−9.60
C05789/HMDB04157	L-Urobilinogen	Tetrapyrroles and derivatives	Porphyrin and chlorophyll metabolism: Bilirubin degradation	597.3633	12.72	−6.60	−4.69	−8.13
C18170/HMDB12880	3-Acetamidopropanal	Carbonyl compounds	Spermine and spermidine degradation Polyamine metabolism	114.0562	0.51	+3.57	+3.27	−13.06
C00873/HMDB61196	Angiotensin I	Polypeptides	Renin-angiotensin system	1318.6613	22.50	+17.68	+14.72	+11.76
C17935	Cysteinyldopa	−	Tyrosine metabolism	317.0817	0.67	+6.54	+8.32	−8.28
C17240	p-Hydroxybenzyl-desulphoglucosinolate	−	Glucosinolate biosynthesis 2-Oxocarboxylic acid metabolism	346.0939	1.20	−2.41	+1.35	−14.42
C06428/HMDB01999/LMFA01030761	Eicosapentaenoic acid [EPA (d5)]	Fatty acyls	Biosynthesis of unsaturated fatty acids	330.2456	15.55	−7.59	−8.13	−15.94
C16531	Docosenoyl-CoA	Fatty acyls	Biosynthesis of unsaturated fatty acids	1088.4354	14.69	−3.94	−11.05	−10.94
C02939/HMDB01113	Isovaleryl-CoA	Fatty acyls	Valine, leucine and isoleucine degradation	874.1622	0.63	−10.55	+5.22	−14.14
C14827/LMFA02000012	9(S)-HpODE	Fatty acyls	Linoleic acid metabolism	311.2229	16.66	+4.12	+10.26	−8.32
C04717/LMFA02000034	13(S)-HpODE	Fatty acyls	Linoleic acid metabolism	335.2206	13.73	+1.52	−3.24	+2.96
C16318/LMFA02020016	methyl (+)-7-isojasmonate	Fatty acyls	alpha-Linoleic acid metabolism	223.1337	15.58	+1.89	+5.78	-8.27
C05984/HMDB00008	DL-a-Hydroxybutyric acid	Fatty acyls	Propanoate metabolism	103.0403	0.61	+0.57	−0.26	−19.02
C01120/HMDB01383/LMSP01050002	Sphinganine 1-phosphate	Sphingolipids	Sphingolipid metabolism	382.2716	16.16	−10.38	−17.50	−17.50
C06124/LMSP01050001	Sphingosine-1-phosphate	Sphingolipids	Sphingolipid metabolism	378.2405	15.51	−14.12	−14.13	−2.72
C12144/HMDB04610/LMSP01030001	Phytosphingosine	Sphingolipids	Sphingolipid metabolism	318.3004	13.94	−5.91	−12.93	−17.48
C03033/HMDB10320	Cortolone-3-glucuronide	Sterol lipids	Estrogen metabolism	543.2802	10.63	+3.49	+2.33	−10.54
C05464/HMDB00631	Glycodeoxycholate	Sterol lipids	Secondary bile acid biosynthesis	450.3207	14.86	+4.81	−4.45	−2.52
C05122/HMDB00036/LMST05040001	Taurocholic acid	Sterol lipids	Primary and Secondary bile acid biosynthesis	533.3256	12.03	+7.73	+8.27	−1.35
C05465/HMDB00951/LMST05040005	Taurochenodeoxycholic acid	Sterol lipids	Primary and Secondary bile acid biosynthesis	500.3040	13.60	+2.64	+2.92	−3.82
C03033/HMDB02579/LMST05010048	Glycochenodeoxycholic acid 3-glucuronide	Sterol lipids	Pentose and glucoronate interconversions Starch and sucrose metabolism Bile secretion	626.3539	12.97	−5.68	−17.01	−17.01
C03033/HMDB10351	11-beta-hydroxyandrosterone-3-glucuronide	Sterol lipids	Pentose and glucoronate interconversions Starch and sucrose metabolism Bile secretion	481.2431	9.22	+4.18	+3.50	−11.59
C03033/HMDB10357	Tetrahydroaldosterone-3-glucuronide	Sterol lipids	Pentose and glucoronate interconversions	558.2907	13.37	+6.04	−7.40	−11.88
			Starch and sucrose metabolism					
			Bile secretion					
C03642/HMDB02580/LMST05020003	Taurolithocholic acid 3-sulfate	Sterol lipids	Bile secretion	562.2492	11.66	+0.53	−14.56	−14.56
C05300/HMDB00335/LMST02010041	16alpha-Hydroxyestrone	Sterol lipids	Steroid hormone biosynthesis	287.1655	12.57	−6.01	−6.01	−6.01
C11136/LMST05010014	Etiocholan-3α-ol-17-one 3-glucuronide	Sterol lipids	Steroid hormone biosynthesis	465.2477	11.09	+2.20	−3.61	−14.8
C18043/HMDB00653/LMST05020016	Cholesterol sulfate	Sterol lipids	Steroid hormone biosynthesis	465.3037	21.47	−8.71	+1.65	−4.43
C18044/HMDB00774/LMST05020014	Pregnenolone sulfate	Sterol lipids	Steroid hormone biosynthesis	395.1891	13.20	−1.32	+12.43	−1.32
C05473/LMST02030187	11b,21-Dihydroxy-3,20-oxo-5b-pregnan-18-al	Sterol lipids	Steroid hormone biosynthesis	363.2171	11.08	−5.78	−9.27	−8.89
C05471/HMDB03259/LMST02030204	Dihydrocortisol	Sterol lipids	Steroid hormone biosynthesis	365.2318	10.68	+0.83	+2.46	−6.44
C05683/	2-Aminoethylphosphocholate	Sterol lipids	Phosphonate and phosphinate metabolism	516.3065	17.26	+4.70	+16.68	−
C03428/HMDB01278/LMPR0106010003	Presqualene diphosphate	Prenol lipids	Cholesterol biosynthesis	604.3531	8.34	+7.97	+8.54	+3.66

a *Expression level indicates log_2_ fold change (FC). + indicates up-regulated in post-eradication group; − indicates down-regulated in post-eradication group*.

When plasma metabolome from Baseline was compared with post-*H. pylori* eradication groups, a number of fatty acids and sphingolipids involved in biosynthesis of unsaturated fatty acids and sphingolipid metabolism respectively were down- regulated post-*H. pylori* eradication. Two metabolites, melatonin and 5-Hydroxytryptophan, which are involved in the melatonin biosynthesis were also found to be down-regulated in post-*H. pylori* eradication groups. Also, propinol adenylate (propanoate metabolism), adenosylcobalamin (riboflavin metabolism) as well as UDP L-rhamnose, and 2,4-Bis(acetamido)-2,4,6-trideoxy-beta-L-altropyranose (amino sugar and nucleotide sugar metabolism) were also down-regulated in post-*H. pylori* eradication groups. Conversely, pseudouridine, oxytocin, angiotensin I, and prequalene diphosphate, involved in pyrimidine metabolism, cAMP signaling pathway, renin-angiotensin system, and cholesterol biosynthesis respectively, were found to be up-regulated following *H. pylori* eradication. Taurocholic acid and taurochenodeoxycholic acid which are involved in primary bile acid biosynthesis were up-regulated at 6 and 12 months-post eradication but they were down-regulated at 18 months-post eradication. Interestingly, there were five metabolites that belong to the class of tetrapyrroles and derivatives which are involved in bilirubin degradation down-regulated at the 12 and 18 months-post *H. pylori* eradication. Besides, four porphyrins which also belong to the class of tetrapyrroles and derivatives that involved in heme biosynthesis were also found to be down-regulated at 18 months post-eradication. Metabolites involved in tricarboxylic acid cycle (TCA), citric acid and flavin adenine dinucleotide (FAD), were found to be up-regulated 6 months post-eradication but they were down-regulated at 12 and 18 months post-eradication. N1,N12-Diacetylspermine and 3-Acetamidopropanal are intermediates that involved in polyamine metabolism. Both of these metabolites were up-regulated at 6 and 12 months post-*H. pylori* eradication. At 18 months post-eradication, N1,N12-Diacetylspermine remained unchanged but 3-Acetamidopropanal was found to be down-regulated (Table 2).

## Discussions

### Biological significance of the fecal lipidomics analysis

Fecal lipidomics analysis allows us to assess the human GI functions broadly and non-invasively and hence, it should reflect the GI health and functions (Gregory et al., 2013). Lipids are essential to intestinal biology as it is more stable than various metabolites, and more conserved across microbiota (Gregory et al., 2013). A series of lipids, which could elucidate the local effects of *H. pylori* eradication on the gut microbiota and modulation of energy were identified.

The endocannabinoid system is a vital endogenous signaling system that comprises of the cannabinoid receptors, their endogenous ligands (the endocannabinoids), and the enzymes catalyzing anabolism and catabolism of endocannabinoid (Di Marzo et al., 2004). This system is recognized to be involved in a extensive range of physiological functions and pathophysiological conditions (Engeli et al., 2005; Pacher et al., 2006; Di Marzo, 2008; Engeli, 2008; Pataky et al., 2013). Obesity is characterized by altered gut microbiota, low grade inflammation and dysregulation of endocannabinoid system, in majority of the cases overactive of the system (Muccioli et al., 2010; Geurts et al., 2011). In this study, Anandamide 0-phosphate was found to be up-regulated at 6 and 12 months following *H. pylori* eradication but it was then down-regulated at 18 months post-eradication. Anandamide 0-phosphate is one of the intermediates in the biosynthesis of anandamine (KEGG: map04723). Anandamide (also known as *N*-arachidonoylethanolamine or AEA) is an endocannabinoid that is synthesized from N-arachidonoyl phosphatidylethanolamine (NAPE) by numerous pathways or alternatively, it can also be synthesized from free arachidonic acid and ethanolamine by the reversal action of a fatty acid amide hydrolase (FAAH) (Sugiura, 2008; Wang and Ueda, 2009). The marked changes in expression of Anandamide 0-phosphate could be linked with the perturbation of gut microbiota post-*H. pylori* eradication which in turn causes dysregulation of the endocannabinoid system and subsequently affect the regulation of energy metabolism and therefore, lead to the development of obesity.

Phytosphingosine, sphinganine and ceramide that are involved in sphingolipid metabolism (KEGG: map00600) were found to be changed significantly in this study. Ceramide is a main molecule in sphingolipid metabolism and a precursor of complex sphingolipids (Sugiura et al., 2002; Hannun and Obeid, 2008). Sphingolipids are important signal molecules that mediate several biological functions for instance cell proliferation, inflammation, and apoptosis (Futerman and Hannun, 2004; Hannun and Obeid, 2008; Morad and Cabot, 2013). Phytosphingosine detected in fecal samples was down-regulated post-*H. pylori* eradication and we believe that it could be related to the reduced inflammation of the stomach lining after *H. pylori* eradication. On the contrary, sphinganine (also known as dihydrosphingosine) detected in the fecal samples was found to be up-regulated significantly 6 and 12 months post-eradication. The biological significance of this conflicting observation was unknown.

One of the most interesting findings of this study is Lipid A-disaccharide-1-P was significantly elevated 12 and 18 months post-*H. pylori* eradication. Lipid A, one of the three structural components of the lipopolysaccharide (LPS) causes the pathophysiological effects associated with Gram-negative bacteria infections (Lodowska et al., 2007). Gram-negative bacteria such as *Bacteroidetes* and *Proteobacteria* (including *H. pylori*) are dominant microorganisms in the gut (Ley et al., 2005; Yap et al., 2016). The up-regulation of Lipid A 12 and 18 months post-*H. pylori* eradication could probably be associated with the perturbation of microbiota in the gut following *H. pylori* eradication. In our previous fecal metagenomics study, we found a transient loss of *H. pylori* instantaneously following *H. pylori* eradication but reappeared at 12–18 months (Yap et al., 2016). Due to the overlapping time points, it is likely that *H. pylori* employs the advantage of ecological niche to replicate intracellularly and survive the anti-bacterial treatment (Yap et al., 2016). Nevertheless, we could not prove that the Lipid A was from *H. pylori*.

A *Proteobacteria* hopanoid named 32,35-anhydrobacteriohopaneterol (Talbot et al., 2007) was found to be down-regulated at 18 months post-eradication. Hopanoids are bacterial pentacyclic triterpenoids that are analogous to eukaryotic steroids structurally and biosynthetically (Ourisson et al., 1987; Doughty et al., 2011; Welander et al., 2012), but their cellular roles are poorly understood (Doughty et al., 2014). Hopanoids may play a role in the alteration of cell membrane permeability in adaptation to extreme environmental conditions in many bacteria. They are formed in the aerial hyphae of the prokaryotic soil bacteria *Streptomyces* which is believed to minimize water evaporation across the membrane (Poralla et al., 2000). In the actinomycete *Frankia*, the hopanoids in diazovesicle membranes probably restrict the entry of oxygen by building the lipid bilayer tighter and more compact (Berry et al., 1993). The *Proteobacteria* in the gut including *H. pylori* may be expressing 32,35-anhydrobacteriohopaneterol to adapt the hostile environment in the gut. The perturbation of gut microbiota following *H. pylori* eradication therapy may be the reason of the reduced expression of this hopanoid.

### Biological significance of the plasma metabolomics analysis

Plasma metabolites is an integrative biofluid that incorporates the functions and phenotypes of many different parts of body in one sample and hence provide an overview of “metabolic footprint” of many areas of metabolism in the human body (Dunn et al., 2011). The identified potential small molecules detected cover the essential metabolic pathways, hence allowing the determination of key intermediates of heme biosynthesis and bilirubin degradation, amino acid metabolisms, lipid metabolism, and energy metabolism (tricarboxylic acid cycle). Numerous metabolites in the plasma were significantly changed following *H. pylori* eradication, with the majority being down-regulated in the post-*H. pylori* eradication groups. A sophisticated interaction of how the changes in the gut microbiota (local) after *H. pylori* eradication influence the systemic changes of a human body could be observed.

### Altered energy metabolism and its association with metabolic disorders

Melatonin and 5-Hydroxytryptophan involved in melatonin biosynthesis were down-regulated following *H. pylori* eradication. Recent experimental evidence suggested that melatonin may influence food intake, energy expenditure, the accumulation of energy in adipose tissue, insulin secretion and glycemic control (Peschke et al., 2006; Picinato et al., 2008; Prokopenko et al., 2009; Amaral et al., 2014b). It has been proposed that the reduction in melatonin production may induce insulin resistance, glucose intolerance, sleep disturbance, and metabolic circadian disorganization characterizing a state of chrono-disruption leading to metabolic disorders such as obesity (Pulimeno et al., 2013; Amaral et al., 2014a; Cipolla-Neto et al., 2014; Sharma et al., 2015). Taken together, the down-regulation of 5-Hydroxytryptophan and melatonin in plasma post-*H. pylori* eradication may be associated with the protective effect of *H. pylori* against metabolic disorders.

6-phosphogluconic acid, in the pentose phosphate pathway, was elevated at 6 months post-eradication. This finding is in agreement with that of a previous study on hepatic gene expression whereby it was reported that the pentose phosphate pathway was up-regulated in obese patients with type 2 diabetes (Takamura et al., 2008). An *in vivo* study also demonstrated significance increase of G6PD expression and activity, NADPH levels, and 6-phosphogluconic acid generation in the liver of adult male Zucker fa/fa rats, a prototype model of hyperglycemia and type 2 diabetes (Gupte et al., 2009). Therefore, the elevation of 6-phosphogluconic acid at 6 months post-eradication could be a metabolic signature of metabolic disorders. However, it seems likely the expression of this metabolite was restored to Baseline level at 12 and 18 months post-eradication.

N-Methyl-4-pyridone-3-carboxamide is one of the end products of nicotinamide-adenine dinucleotide (NAD) degradation. This metabolite was initially up-regulated at 6 months post-eradication but it was down-regulated at 12 and 18 months post-eradication. NAD is one of the cofactors essential for redox reactions. NAD and NADP play pivotal roles in metabolic conversions as signal transducers and in cellular defense systems in which they participate as electron carriers in energy transduction and biosynthetic processes (Pollak et al., 2007). This observation indicated that *H. pylori* eradication may affect the biosynthesis of NAD in nicotinate and nicotinamide metabolism and hence, it corroborated with our previous finding that *H. pylori* eradication may affect the regulation of energy metabolism in the human body (Yap et al., 2015).

Citric acid and flavin adenine dinucleotide (FAD), an intermediate and a cofactor of the tricarboxylic acid cycle (TCA), were changed post-*H. pylori* eradication. In human beings, the TCA cycle together with oxidative phosphorylation, provides more than 95% of energy used by aerobic cells (Berg et al., 2002). In addition, accumulating evidence has emphasized the vital roles of gut microbiota in energy harvest and metabolism in hosts (Bäckhed et al., 2004; Musso et al., 2010; Velagapudi et al., 2010; Venema, 2010), which are thought to be associated with various diseases such as obesity and diabetes. The down-regulation of these metabolites at 12 and 18 months post-eradication could probably reflect the long term effect of *H. pylori* eradication on regulation of energy metabolism in which the eradication of this bacterium causes the perturbations of the gut microflora and therefore, it affects regulation of energy metabolism and subsequently leads to development of metabolic disorders such as obesity. This result could be an example to demonstrate the role of gut bacteria on plasma metabolite profile.

N1,N12-diacetylspermine is an acetylated product of spermidine and spermine controlled by spermidine-spermineN1-acetyltransferase (SSAT) using acetyl-CoA as substrate. Polyamines (putrescine, spermine and spermidine) are organic polycations that are important for various cellular functions such as cell growth, cancer and aging of which its metabolism is tightly controlled (Pegg and Casero, 2011). Both *in vivo* and *in vitro* studies have shown that polyamine pathway plays a major role in energy homeostasis (Jell et al., 2007; Kraus et al., 2014). The up-regulation of N1,N12-diacetylspermine at 6 and 12 months post-eradication but with no significant changes at 18 months-post eradication may indicate the dysregulation of polyamine metabolism which could be a potential predictive signature of metabolic disorders such as obesity.

Studies also showed that the increase in blood concentrations of selected essential amino acids and their derivatives, in particular, branched-chain amino acids (BCAA), aromatic amino acids (Adams, 2011), and the decreases in the metabolism of essential fatty acids (linoleic and α-linoleic acids) and polyunsaturated fatty acids [such as eicosapentaenoic acid (EPA)] (Das, 2006), are believed to play an important role in the pathophysiology of several diseases including obesity. Correspondingly, in this study, we found that a cofactor involved in the metabolism of BCAA, isovaleryl-CoA, as well as numerous small molecules involved in aromatic amino acid metabolisms (tryptophan, tyrosine and phenylalanine metabolisms) were perturbed following *H. pylori* eradication and these small molecules were all down-regulated at 18 months-post eradication. The decrease in the metabolisms of BCAA and aromatic amino acids following *H. pylori* eradication could indirectly lead to the accumulation of these amino acids in the blood. The intermediates involved in the essential fatty acid metabolisms (linoleic and α-linoleic acids metabolism) and the polyunsaturated fatty acid EPA were down-regulated at 18 months post-*H. pylori* eradication as well. Therefore, these dysregulated fatty acid and amino acid metabolisms may be associated with the future onset of metabolic disorders.

Following *H. pylori* eradication was the down-regulation of tetrapyrroles and derivatives that are involved in heme biosynthesis and bilirubin degradation. Coproporphyrinogen III, protoporphyrin IX, hematoporphyrin, and harderoporphyrin are naturally occurring porphyrins, intermediates that play an important role in heme biosynthesis. Heme is critical also for the biological functions of several enzymes, such as cytochromes (Ajioka et al., 2006). The down-regulation of porphyrins is another observation that may support our previous findings that *H. pylori* eradication may affect the regulation of energy metabolism and hence, possibly lead to the development of metabolic disorders such as obesity (Yap et al., 2015).

The synthesis of bile acids (BAs) is the key pathway of cholesterol catabolism in human. BAs are now regarded as metabolic integrators of whole-body energy homeostasis (Du et al., 2013). BAs can influence glucose and lipid metabolism through the activation of farnesoid X receptor (FXR), lowers plasma triglyceride (TG) synthesis and/or the modulation of glucose-induced lipogenic genes (Lefebvre et al., 2009). Furthermore, previous studies have shown that the importance of intestinal bacteria on the metabolism of BAs (Cummings and Macfarlane, 1997) and modulation of lipid metabolism (Velagapudi et al., 2010). Up-regulation of prequalene diphosphate, an intermediate involved in cholesterol biosynthesis, was probably linked to the increase of bile acid in the post-*H. pylori* eradication plasma. We also identified two BAs, taurocholic acid and taurochenodeoxycholic acid in which they were up-regulated at 6 and 12 months post-*H. pylori* eradication but down-regulated at 18 months-post eradication. Consistently, majority of the TG in our study were found to be down-regulated at 6 and 12 months post-*H. pylori* eradication (File S1). Hence, the elevation of BAs in the plasma could also be the indication of possibility of future onset of metabolic disorders. However, at 18 months post-eradication, the bile acids in plasma were down-regulated instead, and strange enough, majority of the plasma TG were still down-regulated as compared to Baseline.

### Altered oxidative stress and its association with immunological disorders

The down-regulation of Biliverdin IX, which acts as the precursor of biosynthesis of bilirubin, as well as uribilinoids (Mesobilirubinogen and L-Urobilinogen) and urobilins (I-Urobilin and L-Urobilin), which are by-products of bilirubin degradation could be associated with the down-regulation of cellular antioxidant activity. Bilirubin is a lipophilic linear tetrapyrrole found abundantly in blood plasma of mammals. It is the final product of heme catabolism. The first major step of heme catabolic pathway involves the formation of water-soluble biliverdin from heme by heme oxygenase (HO) which is subsequently reduced by biliverdin reductase (BVR) to bilirubin (Baranano et al., 2002). Studies have shown that bilirubin is a main physiologic antioxidant cytoprotectant (Stocker et al., 1987; Baranano et al., 2002; Kapitulnik, 2004; Sedlak et al., 2009) which can protect cells from a 10,000-fold excess of H_2_O_2_ (Baranano et al., 2002; Sedlak et al., 2009). The down-regulation of biliverdin IX in plasma post-*H. pylori* eradication may indicate low concentration of bilirubin in tissues that leads to marked increase of reactive oxygen species in the tissue levels and causes apoptotic cell death (Baranano et al., 2002; Sedlak et al., 2009).

In addition, it has been reported that oxidative stress may play a crucial role in the pathogenesis of asthma. For instance, Ohrui et al. reported a case of significant relief of asthma symptoms during jaundice (Ohrui et al., 2003). On the other hand, accumulating evidence suggests that bilirubin also possesses immunomodulatory properties (Nejedlá, 1970; Větvička et al., 1985; Haga et al., 1996; Kirkby and Adin, 2006; Liu et al., 2008) and may protect mammals against autoimmune diseases (Liu et al., 2008). Therefore, the down-regulation of Biliverdin IX and by-products of bilirubin following *H. pylori* eradication could be related to the negative association of *H. pylori* with asthma and allergy (Amedei et al., 2006, 2010; Blaser et al., 2008) and autoimmune diseases (Sawalha et al., 2004; Ram et al., 2013) where *H. pylori* may have protective effect against some of the immunological disorders.

### Altered sphingolipid metabolism and its association with inflammation

Ceramide, sphingosine, sphingosine 1-phosphate (S1P), and ceramide-1-phosphate (C1P) are the main bioactive sphingolipids that act as signaling molecules to regulate physiological events such as cell proliferation, apoptosis, and inflammation (Futerman and Hannun, 2004; Hannun and Obeid, 2008; Morad and Cabot, 2013). Ceramide is an important molecule in sphingolipid metabolism (KEGG: map00600) and a precursor of complex sphingolipids. S1P has strong proinflammatory properties. It activates neutrophils and macrophages and further induces mast cells degranulation. S1P also stimulates cyclooxygenase 2 (COX2), thus leading to production of inflammatory mediators (Takeuchi et al., 2006). It is known that colonization of the stomach by *H. pylori* can result in chronic gastritis, and in minority cases, it causes the development of peptic ulcers at the site of colonization (Kusters et al., 2006). Eradicating *H. pylori* can permanently cure of most gastric and duodenal ulcers. In our study, following *H. pylori* eradication, the down-regulation of S1P, sphinganine 1-phosphate and phytosphingosine could probably be related to the reduction of sub-clinical inflammation in the stomach lining. These observations probably complimented with the down-regulation of fecal pytosphingosine post- *H. pylori* eradication. It is important to note that we also observed significant changes of ceramides and other more complex sphingolipids, such as glucosylceramides, and lactosylceramide in the plasma but they did not display a consistent pattern of expression (File S2). Studies have shown that lipid metabolism in blood could be impacted by the changes of gut microbiota (Velagapudi et al., 2010). Thus, changes of expression of sphingolipids in this study may reflect another aspect of the influence of gut microflora on biochemical reactions in blood.

### Others

On filtering out exogenous plasma metabolites that were not produced by the human body, two hopanoids produced by *Proteobacteria* in the plasma, bacteriohopane-,32,33,34-triol-35-cyclitolguanine and 2-methyl-32,35-anhydrobacteriohopanetetrol, were detected that may be correlated with the *Proteobacteria* hopanoid (32,35-anhydrobacteriohopaneterol) found in fecal lipidome. Similar to the hopanoid found in fecal lipidome, these two plasma hopanoids were also down-regulated post-*H. pylori* eradication (File S3). This observation was yet again another instance that indicates changes in the gut microbiota (local) impact the systemic changes of human body following *H. pylori* eradication.

Beside the loss of *H. pylori*, proton pump inhibitors and antibiotics may also influence the human gut microbiome, fecal lipidome, and plasma metabolome. Several studies had demonstrated that proton pump inhibitor causes significant shifts in microbiome composition, diversity and function (Bajaj et al., 2014; Imhann et al., 2016; Jackson et al., 2016). However, the long-term effects proton pump inhibitors are unknown. The effects of antibiotic therapy on the gut microbiome defers among individuals and antibiotics used (Jakobsson et al., 2010; Zaura et al., 2015). Zaura et al. (2015) showed that clindamycin and ciprofloxacin can cause severe and long-term impact on the health-associated butyrate-producing microbial community of the gut while amoxicillin treatment only resulted in dissimilarity between baseline and week-1 samples compared to placebo group in feces. The diversity of the microbiota has been reported to recover to resemble the pre-treatment states by the end of 1 year after clarithromycin, metronidazole and omeprazole treatment, the microbiota remained perturbed in some cases for up to 4 years post treatment (Jakobsson et al., 2010). The present study could not definitely attribute these observations to the loss of *H. pylori* as we were unable to rule out completely the influences of triple therapy used for *H. pylori* eradication.

## Conclusions

**Figure 6:**
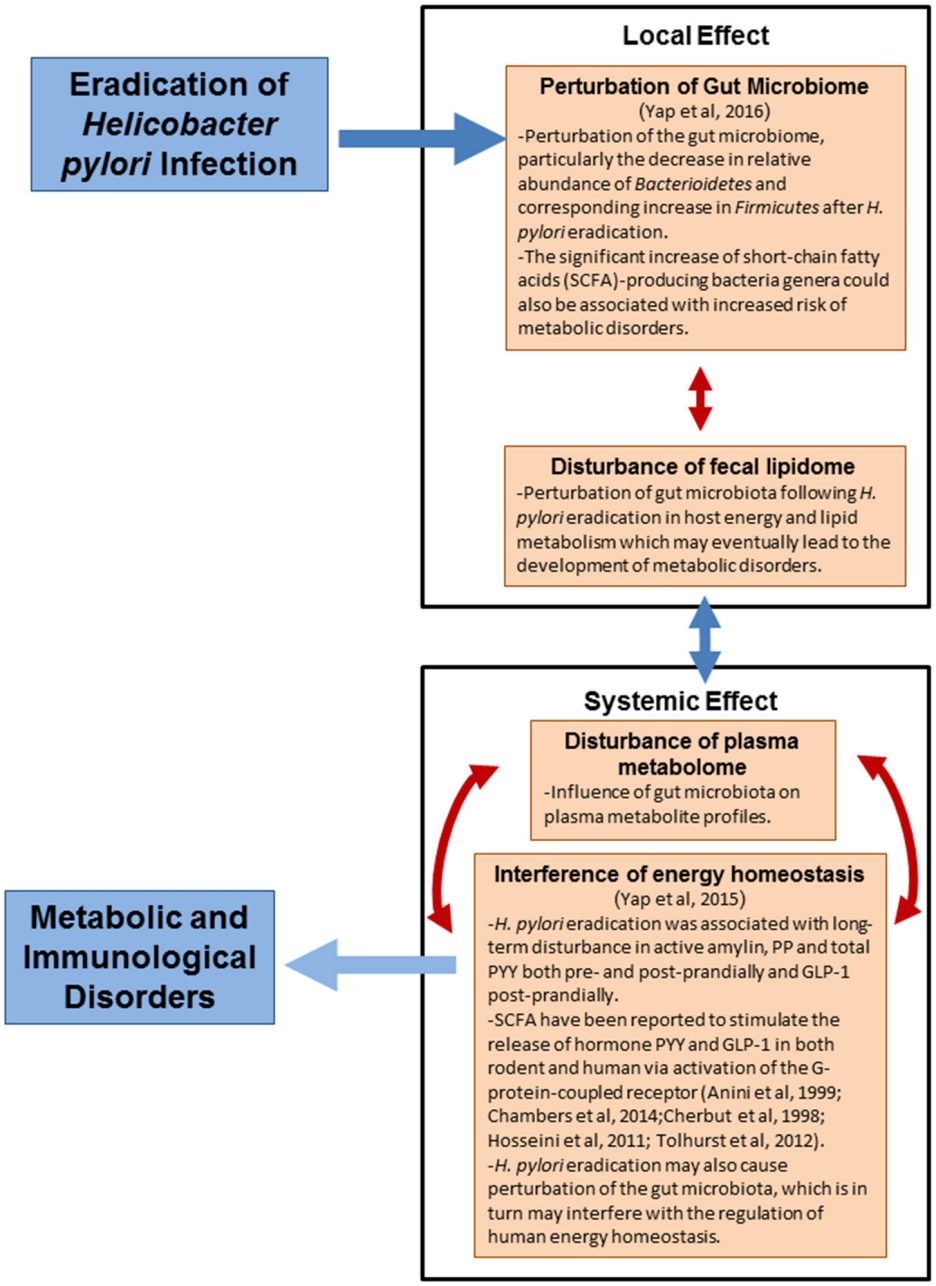
**The local and systemic effects of ***H. pylori*** eradication**.

The local and systemic effects of *H. pylori* eradication and the proposed mechanisms underlying the association of *H. pylori* eradication with human metabolic and immunological disorders are summarized in Figure 6. In conclusion, non-targeted fecal lipidomics and plasma metabolomics revealed that *H. pylori* eradication dramatically changed many global metabolite/lipid features, with the majority of metabolites being down-regulated. We have proposed the influence of gut microbiota on the systemic changes of human body. Our findings primarily implicate the perturbation of gut microbiota following *H. pylori* eradication may affect the energy and lipid metabolism in human which could eventually lead to the development of metabolic disorders. The predictive metabolic signature of metabolic and immunological disorders following *H. pylori* eradication gave us insights on the intricate and complex interaction of *H. pylori* and gut microbiota in modulating human health and therefore, a point to ponder upon in future management of *H. pylori* infection.

## Availability of data and materials

The datasets supporting the conclusions of this article are included within the article (and its additional files).

## Author contributions

GP, ML, KG, and JV conceived and designed the experiments. TY performed the experiments and analyzed the data. JV contributed reagents/materials/analysis tools. TY, GP, DC, ML, KG, and JV contributed to the writing of the manuscript. TY, AL, and AA carried out the recruitment of volunteers. AL and KG carried out the clinical analysis. ML and JV helped to supervise the laboratory works.

## Funding

This study was supported by the University of Malaya-Ministry of Education (UM-MOE) High Impact Research (HIR) Grant UM.C/HIR/MOE/13/4 (HIR Account No: H-50001-00-A000029), University of Malaya Research Grant (UMRG) RP016B-13HTM, the Diane Belfer Program for Human Microbial Ecology, the C, and D fund (anonymous donors), and the Knapp Family Fund.

### Conflict of interest statement

The authors declare that the research was conducted in the absence of any commercial or financial relationships that could be construed as a potential conflict of interest.

## Supplementary material

Figure S1 **Typical LC-ESI-TOF total ion chromatogram of fecal lipid extract from (A)** Baseline, **(B)** 6 months, **(C)** 12 months, and **(D)** 18 months post-eradication group, acquired under ESI positive ionization.

Figure S2 **Typical LC-ESI-TOF total ion chromatogram of fecal lipid extract from (A)** Baseline, **(B)** 6 months, **(C)** 12 months, and **(D)** 18 months post-eradication group, acquired under ESI negative ionization.

Figure S3 **Typical LC-ESI-TOF total ion chromatogram of plasma metabolite extract from (A)** Baseline, **(B)** 6 months, **(C)** 12 months, and **(D)** 18 months post-eradication group, acquired under ESI positive ionization.

Figure S4 **Typical LC-ESI-TOF total ion chromatogram of plasma metabolite extract from (A)** Baseline, **(B)** 6 months, **(C)** 12 months, and **(D)** 18 months post-eradication group, acquired under ESI negative ionization.

Table S1 **Global characterization of the fecal lipidomics data**.

Table S2 **Summary of the differentially expressed fecal lipids**.

Table S3 **Global characterization of the plasma metabolomics data**.

Table S4 **Summary of the differentially expressed plasma metabolites**.

Table S5 **Characteristics of the pooled fecal and plasma sample groups**.

Table S6 **Mixture of Avanti Polar Lipids glycerophospholipid standards**.

File S1 **Significantly changed fecal lipid profiles when Baseline compared with post-*H. pylori* eradication groups**.

File S2 **Significantly changed plasma metabolite profiles when Baseline compared with post-*H. pylori* eradication groups**.

File S3 ***Proteobacteria* hopanoids detected in the plasma metabolite profiles**.
