# Supplementary figures and images for: Global Fecal and Plasma Metabolic Dynamics Related to Helicobacter pylori Eradication
Source: Front Microbiol. 2017 Mar 30;8:536. doi: 10.3389/fmicb.2017.00536 (PMC5371670; doi:10.3389/fmicb.2017.00536)

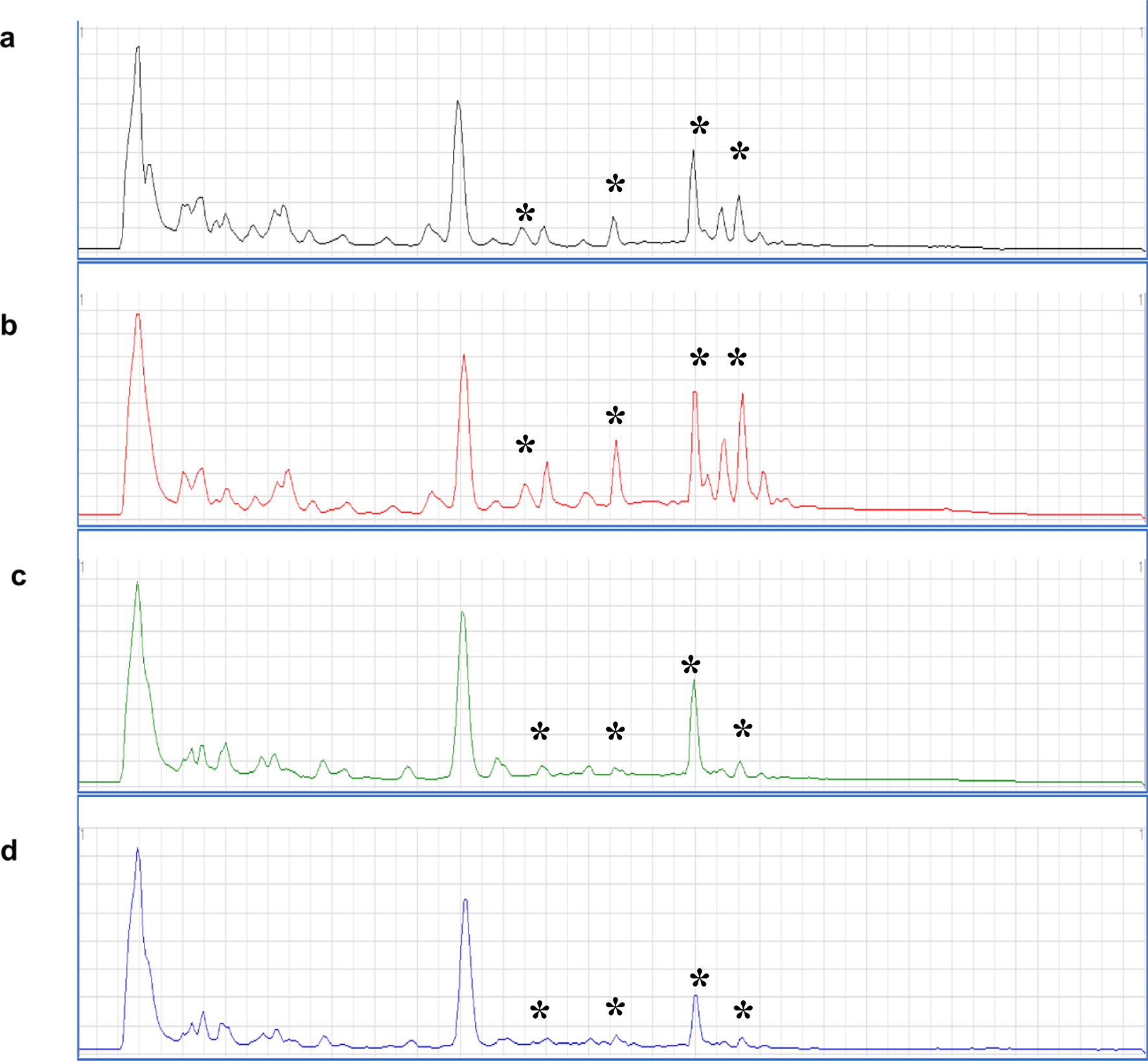

Supplement: Figure S1 — Typical LC-ESI-TOF total ion chromatogram of fecal lipid extract from (A) Baseline, (B) 6 months, (C) 12 months, and (D) 18 months post-eradication group, acquired under ESI positive ionization. [file Image1.TIF]

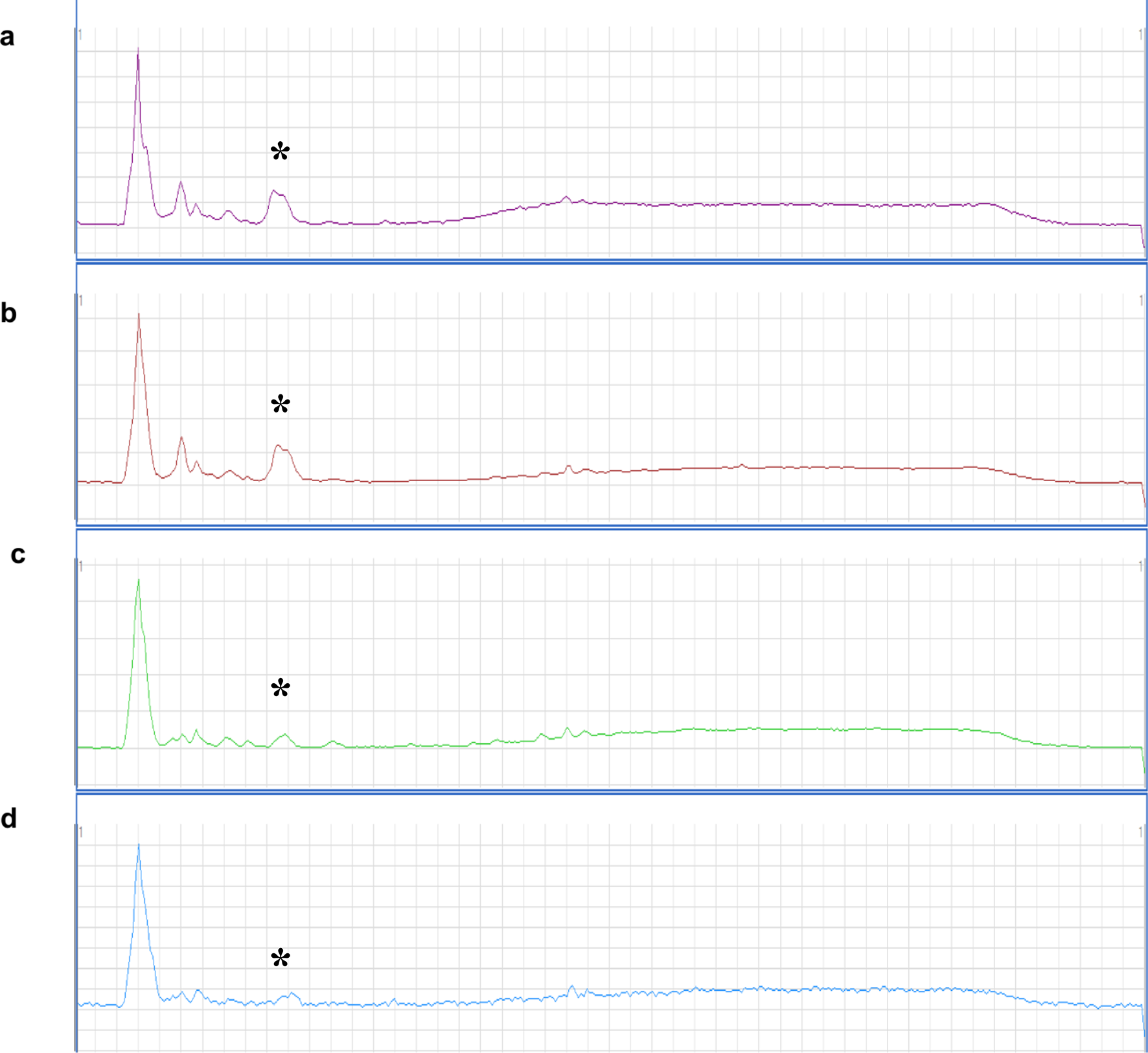

Supplement: Figure S2 — Typical LC-ESI-TOF total ion chromatogram of fecal lipid extract from (A) Baseline, (B) 6 months, (C) 12 months, and (D) 18 months post-eradication group, acquired under ESI negative ionization. [file Image2.TIF]

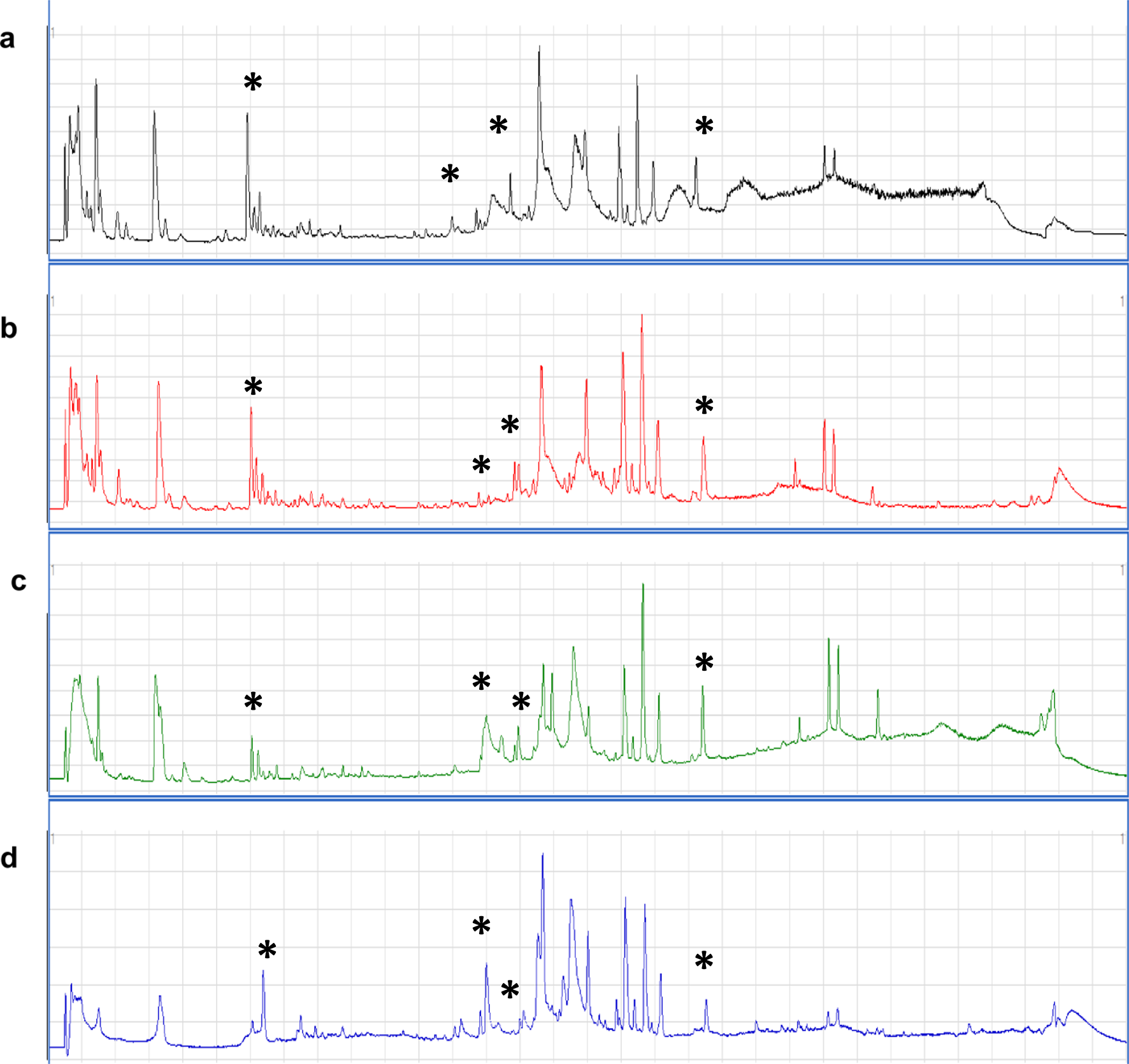

Supplement: Figure S3 — Typical LC-ESI-TOF total ion chromatogram of plasma metabolite extract from (A) Baseline, (B) 6 months, (C) 12 months, and (D) 18 months post-eradication group, acquired under ESI positive ionization. [file Image3.TIF]

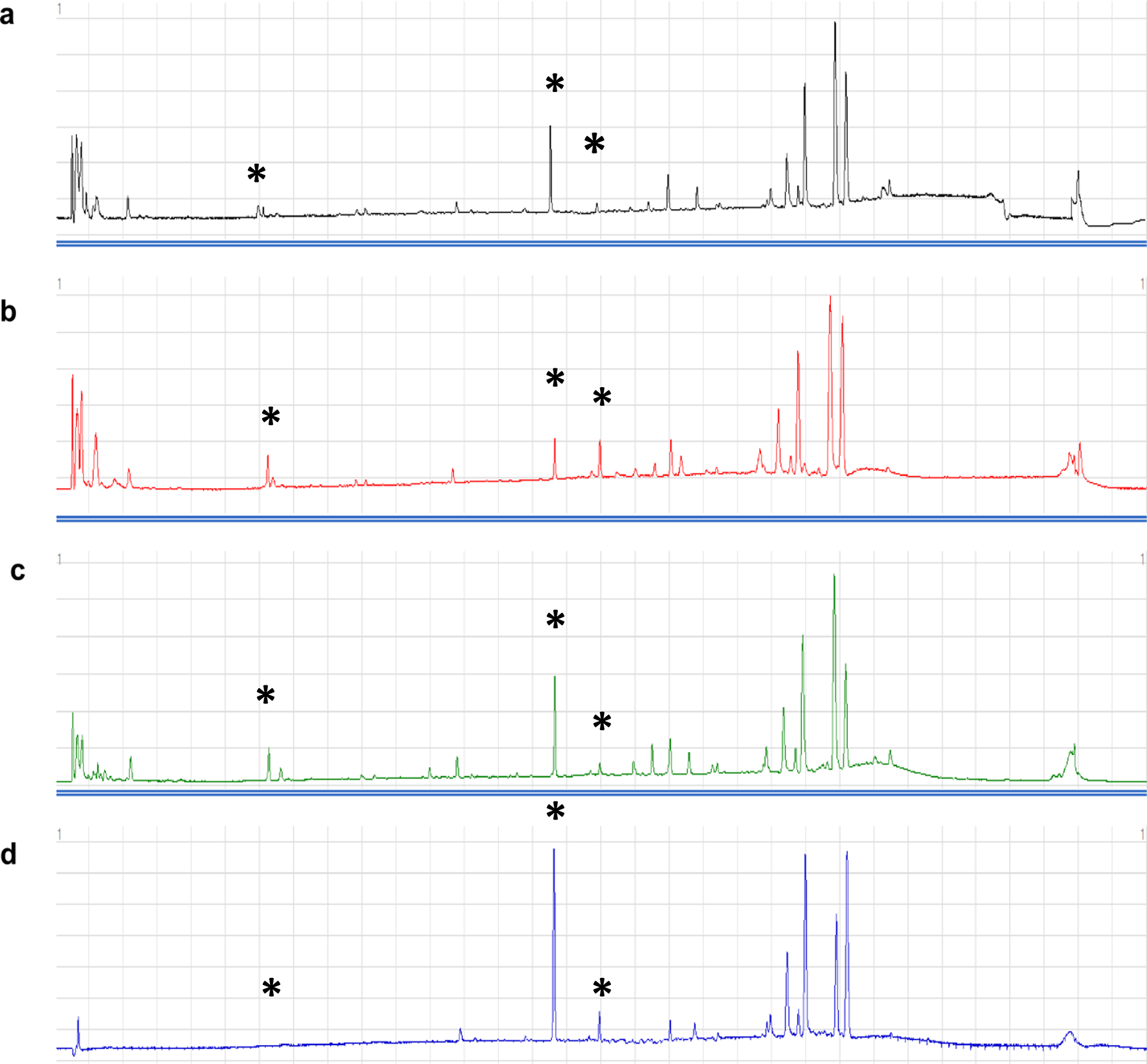

Supplement: Figure S4 — Typical LC-ESI-TOF total ion chromatogram of plasma metabolite extract from (A) Baseline, (B) 6 months, (C) 12 months, and (D) 18 months post-eradication group, acquired under ESI negative ionization. [file Image4.TIF]
