# Supplementary material for: Global Fecal and Plasma Metabolic Dynamics Related to Helicobacter pylori Eradication
Source: Front Microbiol. 2017 Mar 30;8:536. doi: 10.3389/fmicb.2017.00536 (PMC5371670; doi:10.3389/fmicb.2017.00536)
Supplement: Table S1 — Global characterization of the fecal lipidomics data. [file Table1.DOCX]

**Table S1. Global characterization of the fecal lipidomics data.**

| **Group** | **LC-ESI** | **Total** | **Total significantly** | **Total decreased** | **Total increased** |
| --- | --- | --- | --- | --- | --- |
|  | **ionization** | **features** | **altered features^§^** | **features*** | **features*** |
|  | **mode** |  |  |  |  |
| **Baseline** | + | 1686 | - | - | - |
|  | - | 1447 | - | - | - |
| **6 months-** | + | 1628 | 513 | 180 | 333 |
| **post eradication** | - | 1431 | 434 | 66 | 368 |
| **12 months-** | + | 1473 | 513 | 335 | 178 |
| **post eradication** | - | 1421 | 434 | 289 | 145 |
| **18 months-** | + | 1462 | 513 | 347 | 166 |
| **post eradication** | - | 1316 | 434 | 322 | 112 |

^§^Significantly altered features denote those with more than 2 fold changes, p<0.001, FDR<1%.

*Decreased and increased features are relative to the Baseline group.
