# Supplementary material for: Global Fecal and Plasma Metabolic Dynamics Related to Helicobacter pylori Eradication
Source: Front Microbiol. 2017 Mar 30;8:536. doi: 10.3389/fmicb.2017.00536 (PMC5371670; doi:10.3389/fmicb.2017.00536)
Supplement: Table S2 — Summary of the differentially expressed fecal lipids. [file Table2.DOCX]

**Table S2. Summary of the differentially expressed fecal lipids.**

| **Comparison** | **Total significantly** | **Total down-regulated** | **Total up-regulated** |
| --- | --- | --- | --- |
| **group** | **expressed lipids^§^** | **lipids** | **lipids** |
| **Baseline vs. 6 months** | 47 | 13 | 34 |
| **Baseline vs. 12 months** | 72 | 43 | 29 |
| **Baseline vs. 18 months** | 70 | 45 | 25 |

^§^Significantly expressed metabolites denote those with more than 2 fold changes, p<0.001, FDR<1%.
