# Supplementary material for: Global Fecal and Plasma Metabolic Dynamics Related to Helicobacter pylori Eradication
Source: Front Microbiol. 2017 Mar 30;8:536. doi: 10.3389/fmicb.2017.00536 (PMC5371670; doi:10.3389/fmicb.2017.00536)
Supplement: Table S3 — Global characterization of the plasma metabolomics data. [file Table3.DOCX]

**Table S3. Global characterization of the plasma metabolomics data.**

| **Group** | **LC-ESI** | **Total** | **Total significantly** | **Total decreased** | **Total increased** |
| --- | --- | --- | --- | --- | --- |
|  | **ionization** | **features** | **altered features****^§^** | **features*** | **features*** |
|  | **mode** |  |  |  |  |
| **Baseline** | + | 18179 | - | - | - |
|  | - | 4894 | - | - | - |
| **6 months-** | + | 19323 | 5932 | 4165 | 1767 |
| **post eradication** | - | 4824 | 1867 | 679 | 1188 |
| **12 months-** | + | 13828 | 5932 | 3743 | 2189 |
| **post eradication** | - | 3963 | 1867 | 821 | 1046 |
| **18 months-** | + | 6789 | 5932 | 5340 | 592 |
| **post eradication** | - | 2257 | 1867 | 1670 | 197 |

^§^Significantly altered features denote those with more than 2 fold changes, p<0.001, FDR<1%.

*Decreased and increased features are relative to the Baseline group.
