# Supplementary material for: Global Fecal and Plasma Metabolic Dynamics Related to Helicobacter pylori Eradication
Source: Front Microbiol. 2017 Mar 30;8:536. doi: 10.3389/fmicb.2017.00536 (PMC5371670; doi:10.3389/fmicb.2017.00536)
Supplement: Table S4 — Summary of the differentially expressed plasma metabolites. [file Table4.DOCX]

**Table S4. Summary of the differentially expressed plasma metabolites.**

| **Comparison** | **Total significantly** | **Total down-regulated** | **Total up-regulated** |
| --- | --- | --- | --- |
| **group** | **expressed metabolites^§^** | **metabolites** | **metabolites** |
| **Baseline vs. 6 months** | 570 | 348 | 222 |
| **Baseline vs. 12 months** | 598 | 359 | 239 |
| **Baseline vs. 18 months** | 596 | 551 | 45 |

^§^Significantly expressed metabolites denote those with more than 2 fold changes, p<0.001, FDR<1%.
