# Supplementary material for: Global Fecal and Plasma Metabolic Dynamics Related to Helicobacter pylori Eradication
Source: Front Microbiol. 2017 Mar 30;8:536. doi: 10.3389/fmicb.2017.00536 (PMC5371670; doi:10.3389/fmicb.2017.00536)
Supplement: Table S5 — Characteristics of the pooled fecal and plasma sample groups. [file Table5.DOCX]

| **Group** | **Sample** | **Characteristic** | | |
| --- | --- | --- | --- | --- |
|  | **Size** | **Race** | **Gender** | **Body Mass Index (BMI)** |
|  | **(N)** |  |  | **Classification*** |
| **1** | **3** | Malay | Female | Normal |
| **2** | **2** | Malay | Female | Overweight |
| **3** | **1** | Chinese | Male | Normal |
| **4** | **7** | Chinese | Female | Normal |
| **5** | **1** | Indian | Male | Normal |
| **6** | **2** | Indian | Male | Overweight |
| **7** | **2** | Indian | Female | Underweight |
| **8** | **7** | Indian | Female | Normal |
| **9** | **1** | Indian | Female | Overweight |
| **10** | **1** | Others | Male | Normal |
| **11** | **1** | Others | Male | Overweight |
| **12** | **1** | Others | Female | Normal |

**Table S5. Characteristics of the pooled fecal and plasma sample groups.**

*BMI Classification based on World Health Organization (WHO): Underweight: <18.50 kg/m^2^; Normal: 18.50-24.99 kg/m^2^; Overweight: ≥25.00 kg/m^2^.
