# Supplementary material for: Global Fecal and Plasma Metabolic Dynamics Related to Helicobacter pylori Eradication
Source: Front Microbiol. 2017 Mar 30;8:536. doi: 10.3389/fmicb.2017.00536 (PMC5371670; doi:10.3389/fmicb.2017.00536)
Supplement: Table S6 — Mixture of Avanti Polar Lipids glycerophospholipid standards. [file Table6.DOCX]

| **# No** | **LipidMap** | **Name** | **Volume** | **Quantity** | **Molecular** | **Concentration** | **Formula** | **Exact** | **Parent ion (LIPIDS MAP)** | | | **Ionization** |
| --- | --- | --- | --- | --- | --- | --- | --- | --- | --- | --- | --- | --- |
|  | **ID** |  | **(mL)** | **(ug)** | **Weight** | **(uM)** |  | **Mass** | **[M+H]+** | **[M-H]-** | **[M.Cl]-** | **Mode** |
| LM-1002 | LMGP01010003 | 17:0-20:4 PC | 1 | 10.02 | 796.11 | 12.59 | C_45_H_82_NO_8_P | 795.578 | 796.5547 |  | 830.506 | Positive |
| LM-1103 | LMGP02010004 | 21:0-22:6 PE | 1 | 9.91 | 834.16 | 11.88 | C_48_H_84_NO_8_P | 833.593 | 834.5692 |  | 832.5452 | Positive |
| LM-1202 | LMGP04010006 | 17:0-20:4 PG | 1 | 9.76 | 785.04 | 12.43 | C_43_H_80_NO_10_P | 801.552 |  | 783.4784 |  | Negative |
| LM-1303 | LMGP03010004 | 21:0-22:6 PS | 1 | 9.83 | 878.17 | 11.19 | C_49_H_87_N_2_O_10_P | 877.5833 |  | 876.576 |  | Negative |
| LM-1402 | LMGP10010003 | 17:0-20:4 PA | 1 | 10.62 | 710.96 | 14.94 | C_40_H_74_NO_8_P | 710.4887 |  | 709.4814 |  | Negative |
| LM-1503 | LMGP06010005 | 21:0-22.6 PI | 1 | 10 | 970.26 | 10.49 | C_52_H_92_NO_13_P | 969.631 |  | 951.5479 |  | Negative |
| LM-1601 | LMGP01050002 | 17:1 LPC | 1 | 9.82 | 507.64 | 19.35 | C_25_H_50_NO_7_P | 507.3325 | 508.3398 |  |  | Positive |
| LM-1700 | LMGP10050001 | 13:0 LPA | 1 | 9.8 | 368.4 | 26.59 | C_16_H_33_O_7_P | 368.1964 |  | 367.1891 |  | Negative |
| LM-1804 | LMGP12010005 | 22:1 (3)-14:1 CA | 1 | 10.62 | 368.4 | 26.59 | C_89_H_166_O_17_P_2_ | 1569.16 |  | 1568.1528 |  | Negative |

**Table S6. Mixture of Avanti Polar Lipids glycerophospholipid standards.**
